# Defining and measuring proximity to criticality based on univariate time series

**DOI:** 10.1073/pnas.2526984123

**Published:** 2026-10-28

**Authors:** J. Samuel Sooter, Antonio J. Fontenele, Andrea K. Barreiro, Cheng Ly, Keith B. Hengen, Woodrow L. Shew

**Affiliations:** ^a^Department of Physics, University of Arkansas, Fayetteville, AR 72701; ^b^ Department of Mathematics, Southern Methodist University, Dallas, TX 75205; ^c^ Department of Mathematics and Applied Mathematics, Virginia Commonwealth University, Richmond, VA 23284; ^d^ Department of Biology, Washington University, St. Louis, MO 63130

**Keywords:** criticality, time series, renormalization group, information theory, sleep

## Abstract

Diverse social, engineered, and biological systems are made up of multiple interacting dynamical components; to work properly, certain components must be carefully regulated so that their dynamics avoid instability. However, many desirable properties, like optimized computation and control, emerge close to the tipping point between stability and instability, i.e., near criticality. Claims that such dynamical systems operate near criticality have been controversial because it is difficult to assess proximity to criticality when control parameters are unknown, hard to manipulate experimentally, and fluctuating in response to changing environmental or internal conditions. Moreover, observations of system dynamics are often limited to time series measurements of one or a few components. Here we develop a temporal renormalization group theory for defining criticality for univariate time series models and an information theoretic definition of proximity to criticality that is independent of model parameterization. We apply this theory to order-n autoregressive models resulting in a data-analytic strategy for time-resolved estimates of proximity to criticality based on measured univariate time series. After benchmarking our approach on diverse ground-truth cases, we apply it to recordings of spiking activity in the mammalian brain. Our estimate of proximity to criticality predicts behavior and suggests that brain dynamics shift closer to criticality during wakefulness and shift away during deep sleep.

## Significance

Knowledge of many complex systems comes from time series measurements of one or a few parts of the system. These dynamics depend on many detailed properties; must we keep track every detail to understand these systems, or can we hope for simpler, unified rules? A hypothesized unifying principle is that many aspects of function are governed by how close the system is to criticality—a marginally stable state at the edge of instability. Means of testing this hypothesis based on univariate time series data in a rigorous and time-resolved way have been lacking. Here we report fundamental theory for defining criticality in univariate time series models and practical analysis for measuring proximity to criticality based on univariate time series data.

Science makes sense of the world by inferring models from data and then extracting meaning from those models. Often the observed data are a time series, tracking the dynamics of one or a few variables, and the relevant inferred models are time series models (1, 2). Successful models distill essential features of the data, discarding as many irrelevant details as possible (3–6). A compelling argument for the feasibility of this program comes from the renormalization group (RG) (7–9). Originally developed to understand the macroscopic behavior of systems near a phase transition, RG is a theoretical framework for tracking how models change under a change of scale. Surprisingly, RG tells us that most details do not matter; only a few essential model parameters control its macroscopic behavior. This fact suggests that diverse systems—biological, astrophysical, engineered, etc—could share common governing principles despite vastly different details.

**Fig. 1.**
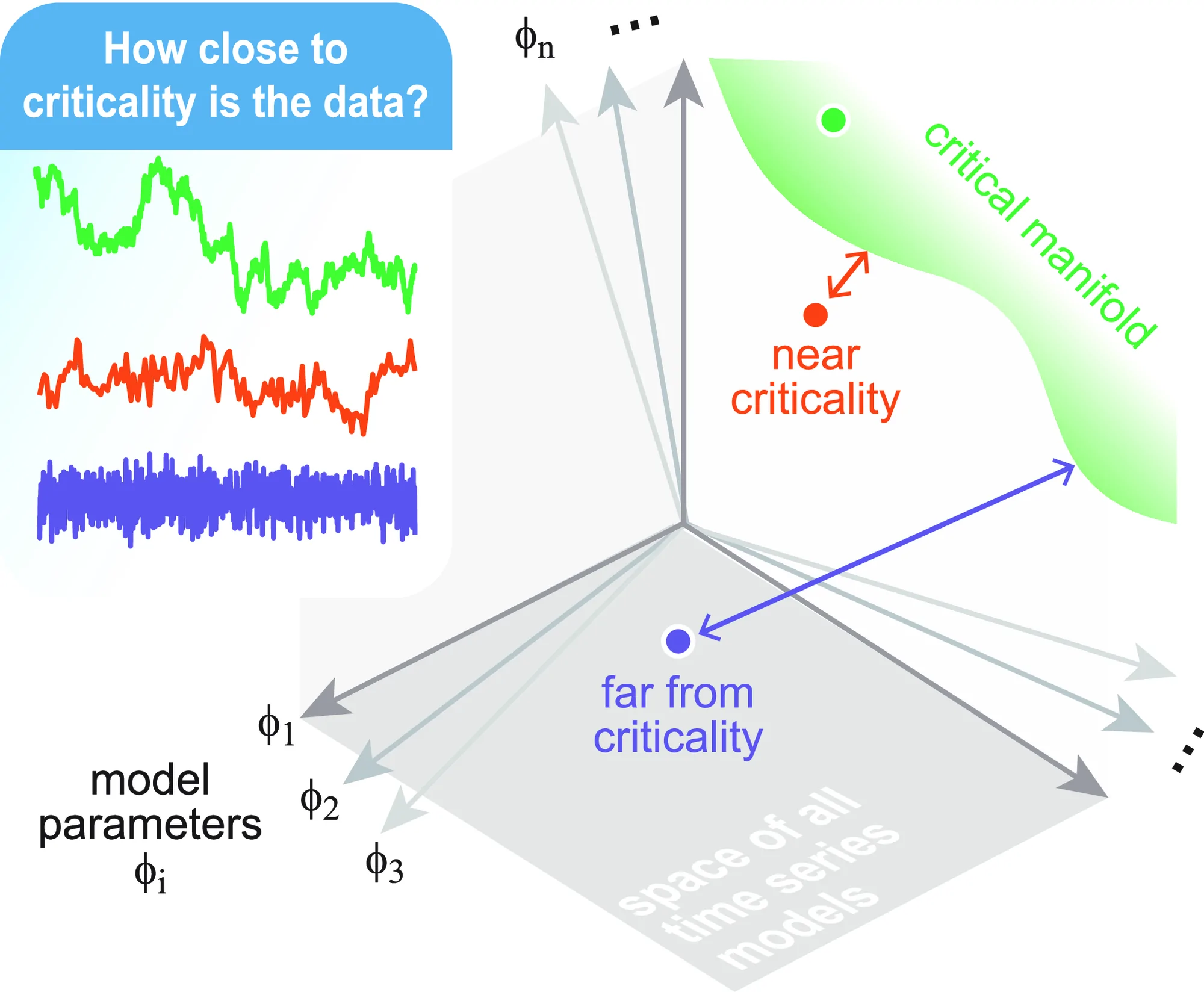
Principled measurement of proximity to criticality for time series. Given a measured univariate time series, we aim to describe the data with a time series model and assess that model’s proximity to criticality without knowledge of the control parameters ($\phi_{i}$) of the system that generated the data. We first develop temporal RG to determine where criticality lies (green) in time series model space. We then use an information theoretic model comparison (KL divergence rate, double-headed arrows) to quantify how far criticality is from a model that best fits the data (filled circles).

From a geometric perspective, RG maps model space—each point in this space is a model and each dimension is a model parameter (Fig. 1) (10–12). RG partitions model space into sets of models sharing the same coarse-scale behavior. For time series models, coarse-scale behavior refers to low-frequency, slow fluctuations. These sets of models are determined by a mapping on model space that transforms a model into a coarse-grained version of itself. Iterating this mapping creates a trajectory, or flow through model space. Fixed points of this mapping are models that are invariant under coarse-graining, i.e. scale-invariant. Excepting “trivial” fixed points (those without correlated fluctuations), the basins of attraction of fixed points usually coincide with boundaries in model space, where small changes in parameters cause large changes in behavior—i.e., phase diagram boundaries. A model lying in one of these basins is at “criticality.”

Theoretical predictions abound for how systems behave at criticality; spatial and/or temporal correlations span an infinite range of scales and power-law dependencies emerge with exponents that obey scaling relations (13–15). Experiments that approximately confirm these predictions have led to claims of criticality in diverse systems from solar flares (16, 17) to social media (18), to flocks of birds (19, 20), to the brain (21–23), and many more. However, these systems have largely unknown phase diagrams and experimentally inaccessible control parameters, which undermines confidence in claims of criticality. This problem is pronounced for biological systems, which self-tune their control parameters in response to their environment. Of particular relevance for how we apply the theory, we develop in this paper is the hypothesis that the mammalian brain operates near criticality, which has important implications for cognition and health (24–26). Observations of approximate power-law fluctuations and predicted scaling laws support this hypothesis (systematically reviewed by ref. 26). The brain self-tunes its state on the fast timescales (seconds-minutes) of neuromodulatory and behavioral state changes (27–31) and on slower timescales (hours-years) of structural plasticity and disease progression (32, 33). To make stronger claims about criticality in the brain, we require a way to track and estimate proximity to criticality without access to control parameters. Here we develop a solution to this challenge that is applicable to univariate time series measured from the brain [e.g., population spike rate (34, 35), a single electroencephalography (EEG) channel (36), or a single functional MRI (fMRI) parcel (37)].

A zoo of ad hoc tools for detecting criticality in neural systems have been proposed (26), most of which are based on analysis of univariate time series. For example, neuronal avalanches (large fluctuations of collective neural activity) are expected to be power-law distributed at criticality (21, 23) and to obey the “crackling noise” scaling relation (11, 38). Thus, deviations from power-law distributions (39–44) and/or scaling relations (23, 28, 32, 33, 45, 46) have been quantified and interpreted as deviation from criticality. Other approaches include branching ratio estimates (21, 47–49), measuring the flatness of an effective potential (50, 51), tracking how statistics change upon phenomenological coarse-graining (22, 52–56), and examining system timescales (24, 57–60). Closest to our approach here, many have fit models to data and examined how best-fit parameters differ from parameters values at criticality (61–64). However, this approach is unsatisfying because it depends on the arbitrary choice of how to parameterize the model.

Here we propose a parameter-free formal definition of distance to criticality, rigorously grounded in RG and information theory. We implement our definition for a fundamental class of univariate time series models—autoregressive (AR) models—which are well suited to fitting univariate experimental data. The result is a method for directly estimating distance to criticality from univariate time series data. This method is applicable in several scenarios. First, certain complex high-dimensional dynamical systems are well described by a univariate mean field model. In such cases, our approach can measure proximity to criticality based on the mean field dynamics (e.g. *SI Appendix*, Fig. S3). In neural systems this occurs when the activity of many neurons, averaged across the population, accounts for a large fraction of the variance of the high-dimensional multivariate system (23, 65–67). Second, a common approach for analyzing multivariate data is to use a dimensionality reduction method to extract univariate time series of interest (68). With such a distilled time series in hand, it may be important to determine if it arises from a dynamical mechanism close to criticality. Our approach offers a principled and practical tool to answer this question.

Below, we first present a formal theory, generally applicable for any univariate time series model. Then we apply the theory to autoregressive models to derive practical data analytic tools. Next, we validate these tools on diverse ground-truth cases and apply them to recordings of population spike count time series measured in mouse and rat brains, making time-resolved estimates of proximity to criticality. Our results suggest that isocortex is closest to criticality while animals are awake and deviates from criticality during sleep.

## 1. Theory

### 1.1. Defining Criticality for Time Series Models: Temporal RG.

Traditionally, RG concerns spatial scale-invariance, because interactions are spatially organized (e.g. near neighbor interactions on an Ising lattice). However, spatial RG is limited for the brain, because neural interactions are largely not spatially organized; <5% of the variance in connection strength among mouse brain regions is explained by their spatial separation (69). Moreover, measurements of brain activity with single-cell resolution are often spatially restricted (usually ∼1 mm^3^). These factors limit empirical assessment of spatial scale-invariance [some notable exceptions include (22, 70–72)]. In contrast, typical measurements are temporally unrestricted, recorded for hours or days with millisecond resolution. What is the appropriate RG scheme for a system in which temporal structure is more accessible than spatial structure?

We consider a univariate time series $x_{1},x_{2},\cdots$ where, for example, $x_{t}$ represents the average activity of a population of neurons in a small patch of cortex at time t. A model for these data is a probability distribution $P(x_{1},x_{2},\cdots )$ for all possible time series. What does it mean for a time series model to be at criticality? To answer this question rigorously, we require an RG program for time series. By direct analogy with Wilson’s momentum space scheme (8), we define a temporal RG (tRG) scheme consisting of the following steps:

1. Switch to frequency domain: Fourier transform $\tilde{x}\left(\omega \right)=\sum_{t}x_{t}e^{-i\omega t}$ the time series to replace $P(x_{1},x_{2},\cdots )$ with $P(\tilde{x})$.
2. Coarse grain: marginalize over Fourier components with frequency above a cutoff $\omega_{0}$. Denoting above-cutoff components by ${\tilde{x}}_{>\omega_{0}}$ and the remaining low-frequency components by ${\tilde{x}}_{\leq \omega_{0}}$, coarse-graining is
  $$\begin{array}{c} P\left({\tilde{x}}_{\leq \omega_{0}}\right)=\int D{\tilde{x}}_{>\omega_{0}}P\left(\tilde{x}\right), \end{array}$$
  where $\int D{\tilde{x}}_{>\omega_{0}}$ denotes functional integration over all possible ${\tilde{x}}_{>\omega_{0}}$.
3. Renormalize: Assessing self-similarity of the original model $P(\tilde{x})$ and the coarse-grained one $P({\tilde{x}}_{\leq \omega_{0}})$ requires rescaling so they have the same range of frequencies,
  $$\begin{array}{c} {\tilde{x}}_{\leq \omega_{0}}\left(\omega \right)\to {\tilde{x}}^{\prime }\left(\omega^{\prime }\right)=\frac{1}{f(\omega_{0})}{\tilde{x}}_{\leq \omega_{0}}\left(\omega_{0}\omega^{\prime }/\pi \right) \end{array}$$
  The rescaled function ${\tilde{x}}^{\prime }$ has the same domain of definition, namely [−π,π], as the original $\tilde{x}$. Writing $P({\tilde{x}}_{\leq \omega_{0}})$ in terms of ${\tilde{x}}^{\prime }$, we obtain the fully renormalized distribution $P({\tilde{x}}^{\prime })$.

**Fig. 2.**
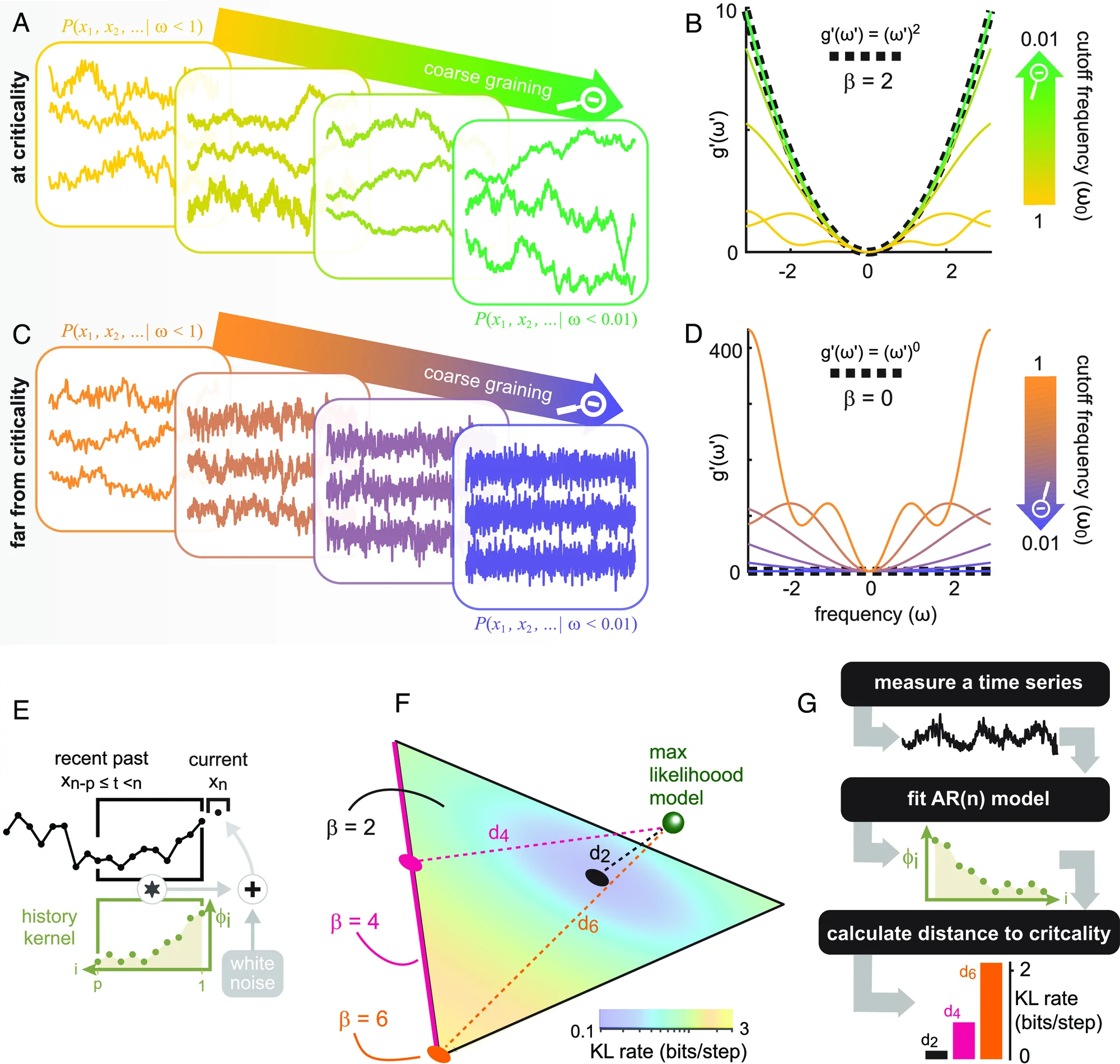
tRG and KL divergence for univariate AR models provides practical tools for estimating proximity to criticality. (*A*) Ensemble of time series generated by a critical AR model (*Left*); applying tRG to this ensemble yields a new ensemble of time series corresponding to a coarse-grained version of the original model (*Right*). (*B*) tRG flow of the AR model in (*A*). Coarse graining, i.e., taking the cutoff frequency $\omega_{0}\to 0$, makes $g^{\prime }\left(\omega^{\prime }\right)\to {\left(\omega^{\prime }\right)}^{2}$ as the model flows into the β=2 fixed point. (*C*) Same as (*A*), but for a non-critical AR model; this model flows into the white noise (β=0) fixed point under tRG. (*D*) Same as (*B*), but for the model in (*C*). (*E*) Anatomy of an AR model. (*F*) The β=2 critical manifold for AR(n) models lies in a patch of an n−1 dimensional hyperplane (in this example, n=3 and the patch is triangular). Along one edge of the β=2 manifold is the (n−2)-dimensional β=4 manifold, along one edge of which is the (n−3)-dimensional β=6 manifold, and so on. Distances to criticality–$d_{2}$, $d_{4}$, and $d_{6}$ - are sketched for an example model (green point). The nearest critical models for each type of criticality are the colored dots on the critical manifold. The surface color of the β=2 manifold indicates the KL divergence rate from the example model to each point on the β=2 manifold. (*G*) Data analytic pipeline: step 1–cquire some time series data, step 2–fit AR(n) model to the data, step 3–use KL divergence rate and our tRG for AR models to measure distances to each tRG fixed point.

A typical time series model has a finite autocorrelation time, say τ. Coarse graining beyond τ (i.e. cutoff frequency $\omega_{0}≲1/\tau$ in step 2) will result in a model without temporal correlations—it flows to the white noise fixed point. Flow to any other fixed point (not white noise) implies temporally correlated scale-invariant dynamics, as discussed in the previous section. Thus, by definition, a time series model that flows into any fixed point, except the trivial white noise fixed point, is at criticality (Fig. 2 *A* and *B*).

### 1.2. Defining Distance to Criticality for Time Series Models: KL Divergence.

Real systems typically have dynamics with a finite autocorrelation time τ and, thus, are not precisely at criticality. However, proximity to criticality matters; system dynamics can change dramatically depending on proximity to criticality. Given a time series model, how close is it to criticality? More specifically, if one model represents some data and another model is at criticality, how does one define distance between the two models in a way that does not depend on an arbitrary choice of model parameterization? Ideally our definition should be based on the model dynamics; two models with very similar dynamics should be “close.” Information theory supplies a natural choice; Kullback–Leibler (KL) divergence (73, 74) per unit time. KL divergence is the unique function of two probability distributions that is continuous, nonnegative, invariant under reparameterization of the distributions, and satisfies a few other properties (74). The KL divergence rate for two models $P_{A}\left(x_{1},x_{2},\cdots \right)$ and $P_{B}\left(x_{1},x_{2},\cdots \right)$ is defined as

$$\begin{array}{cc} & J(P_{A}\left|\right|P_{B}):=\lim_{T\to \infty }\frac{1}{T}\text{KL}(P_{A}\left(x_{1},..,x_{T}\right)\left|\right|P_{B}\left(x_{1},..,x_{T}\right))\\ & =\lim_{T\to \infty }\frac{1}{T}\left(\prod_{t=1}^{T}\int_{-\infty }^{\infty }dx_{t}\right)P_{A}\left(x_{1},..,x_{T}\right)\log_{2}\frac{P_{A}\left(x_{1},..,x_{T}\right)}{P_{B}\left(x_{1},..,x_{T}\right)}, \end{array}$$

where $P_{A}\left(x_{1},\cdots ,x_{T}\right)$ denotes the full model $P_{A}\left(x_{1},x_{2},\cdots \right)$ marginalized over all $x_{t}$ with t>T, and similarly for B.

We now define the distance to criticality of a time series model A as

[1] $$\begin{array}{c} \text{DTC}\left(A\right):=\;\min\{J(P_{A}\left|\right|P_{B}):B\in M_{c}\}, \end{array}$$

where $M_{c}$ is the set of all time series models at criticality (i.e. the set of all models that flow into a nontrivial tRG fixed point). This is the distance from model A to the nearest critical model, where “nearest” means minimal KL divergence rate. This quantity has units of bits per time and can be interpreted as how distinguishable (per unit time) model A is from the nearest critical model. More explicitly, DTC(A) is the expected rate of accumulation of evidence for the hypothesis “the underlying model is A” over the alternative hypothesis “the underlying model is at criticality” when observing a time series generated by A. (We note that KL divergence rate does not adhere to strict mathematical properties of distance metrics; it is not symmetric and it does not obey the triangle inequality. For this reason, we often use the looser term “proximity” instead of distance.)

This definition of DTC is parameter-free (i.e., it depends on the model dynamics, not the model parameters) and has a physically meaningful interpretation (i.e., distinguishability in bits/time). However, working with the set of all critical models $M_{c}$ is infeasible; in practice, we replace $M_{c}$ with its intersection with a tractable family of models M (see *SI Appendix*, section S8 for further discussion). For example, in Section 1.3 we carry out this program analytically with M equal to the family of all univariate autoregressive models of a given order. Section 2 employs our DTC definition in other families of models, including ones that incorporate spiking, nonlinearity, and multiplicative noise.

### 1.3. tRG and DTC for Univariate AR Models.

Measuring DTC from time series data presents two main challenges: i) inferring an unknown model underlying the data and ii) determining the structure of the set $M_{c}$ of critical models. A celebrated approach to challenge i) is maximum entropy modeling (*max ent*) (75). *Max ent* acknowledges that, given limited samples, only low-order statistics, like means and covariances, can be measured accurately. Thus, we seek the minimally presumptive model consistent with these low-order statistics. For time series, the *max ent* model that reproduces measured autocovariances $\hat{C}\left(t\right)$ for lags t=0,1,⋯,n is the order-n AR model (Fig. 2*E*) (76):

$$\begin{array}{c} x_{t}=\sum_{j=1}^{n}\phi_{j}x_{t-j}+\xi_{t}, \end{array}$$

where the history kernel $\phi ={\left(\phi_{1},\cdots ,\phi_{n}\right)}^{T}$ is given by

$$\begin{array}{c} \phi ={\hat{\Gamma }}^{-1}\hat{\gamma } \end{array}$$

and $\xi_{t}$ is Gaussian white noise with variance

$$\begin{array}{c} \sigma^{2}=\hat{C}\left(0\right)-{\hat{\gamma }}^{T}\phi \end{array}$$

Here $\hat{\gamma }={\left(\hat{C}\left(1\right),\cdots ,\hat{C}\left(n\right)\right)}^{T}$ and $\hat{\Gamma }$ is an n×n matrix with ${\hat{\Gamma }}_{\mathrm{jk}}=\hat{C}(|j-k|)$.

In systems with uncontrolled control parameters, like the brain, proximity to criticality is time-dependent. To track such changes, we must measure proximity to criticality in many consecutive short duration periods (e.g. in a sliding window). Considering how few samples are in each short window, AR models are well suited to this endeavor (in the *max ent* sense). We next perform a full tRG analysis of AR(n) models, identifying all the fixed points and their basins of attraction. [RG for AR(2) was previously developed with a different approach (12).] Then, we analytically calculate the KL divergence rate between any two AR models, allowing us to fully realize our rigorous definition of distance to criticality for AR(n) models.

The probability distribution corresponding to an AR model with history kernel ϕ and noise variance $\sigma^{2}$ is (*SI Appendix*, section S4.1)

[2] $$\begin{array}{c} P\left(\tilde{x}\right)\propto \;\exp\left[-\int_{-\pi }^{\pi }d\omega \;g\left(\omega \right){\left|\tilde{x}\left(\omega \right)\right|}^{2}\right], \end{array}$$

where

[3] $$\begin{array}{c} g\left(\omega \right)=\frac{1}{{\left(2\pi \sigma \right)}^{2}}{\left|1-\tilde{\phi }\left(\omega \right)\right|}^{2} \end{array}$$

This makes tRG coarse-graining straightforward; the high and low frequency components factor separately, leaving us with

$$\begin{array}{c} P\left({\tilde{x}}_{<\omega_{0}}\right)=\int D{\tilde{x}}_{\geq \omega_{0}}\;P\left(\tilde{x}\right)\propto \;\text{exp}\left[-\int_{-\omega_{0}}^{\omega_{0}}d\omega \;g\left(\omega \right){\left|\tilde{x}\left(\omega_{0}\right)\right|}^{2}\right] \end{array}$$

After rescaling, this becomes

$$\begin{array}{c} P\left({\tilde{x}}^{\prime }\right)\propto \;\text{exp}\left[-\frac{\omega_{0}}{\pi }|f\left(\omega_{0}\right){|}^{2}\int_{-\pi }^{\pi }d\omega^{\prime }\;g\left(\omega_{0}\omega^{\prime }/\pi \right){\left|{\tilde{x}}^{\prime }\left(\omega^{\prime }\right)\right|}^{2}\right] \end{array}$$

which is the same as the original distribution $P(\tilde{x})$ with g(ω) replaced by

$$\begin{array}{c} g^{\prime }\left(\omega^{\prime }\right)=\frac{\omega_{0}}{\pi }{\left|f\left(\omega_{0}\right)\right|}^{2}g\left(\omega_{0}\omega^{\prime }/\pi \right) \end{array}$$

As $\omega_{0}\to 0^{+}$ (i.e., coarse graining more and more), the behavior of $g^{\prime }\left(\omega^{\prime }\right)$ is determined by the dominant term in its Taylor series expansion, which is an even power of ω because g(ω) is an even function of ω ($\tilde{\phi }\left(-\omega \right)=\tilde{\phi }{\left(\omega \right)}^{*}$). Thus, we can write $g\left(\omega \right)=A\omega^{\beta }+O\left(\omega^{\beta +2}\right)$, where β∈{0,2,4,⋯}. Then choosing $f\left(\omega_{0}\right)=A^{-1/2}{\left(\omega_{0}/\pi \right)}^{-(\beta +1)/2}$ makes

[4] $$\begin{array}{c} P\left({\tilde{x}}^{\prime }\right)\propto \;\text{exp}\left[-\int_{-\pi }^{\pi }d\omega^{\prime }\;{\left(\omega^{\prime }\right)}^{\beta }{\left|{\tilde{x}}^{\prime }\left(\omega^{\prime }\right)\right|}^{2}\right] \end{array}$$

in the $\omega_{0}\to 0^{+}$ limit. We conclude that each value of β corresponds to a different tRG fixed point, and the exponent of the dominant term in g(ω) determines which fixed point an AR model will flow into. Fig. 2 *B* and *D* shows how g(ω) changes upon coarse-graining for two example fixed points.

Determining the sets of AR models that flow into each fixed point then reduces to identifying conditions for the dominant term in g(ω) to have a given exponent. We show in *SI Appendix*, section S4.2 that an AR model flows into the β fixed point (Eq. **4**) for β≥2 if and only if its history kernel ϕ satisfies i) $a_{0}=1$, ii) $a_{m}=0$ for all m∈{1,2,⋯,β/2−1}, and iii) $a_{\beta /2}\neq 0$, where

$$\begin{array}{c} a_{m}=\sum_{t}t^{m}\phi_{t} \end{array}$$

If instead $a_{0}\neq 1$, then the model flows into the trivial β=0 fixed point—the white noise fixed point. The remaining fixed points are nontrivial; models that flow into them exhibit fluctuations across all timescales. In RG parlance, the parameters $a_{0}$, $a_{1}$, ⋯, $a_{\beta /2-1}$ are relevant for the β fixed point. Changing any other details of an AR model, including its noise variance, does not impact its asymptotic behavior at coarse scales. This results in extended basins of attraction of the tRG fixed points in the space of possible history kernels ϕ (Fig. 2*F*).

Now consider a specific AR model, say A. How far is A from criticality? The KL divergence rate between A and another AR model B is (*SI Appendix*, section S4.3)

$$\begin{array}{c} J\left(A||B\right)=\frac{1}{4\pi }\int_{-\pi }^{\pi }d\omega \;\left[\ln\left(\frac{g_{A}\left(\omega \right)}{g_{B}\left(\omega \right)}\right)+\frac{g_{B}\left(\omega \right)}{g_{A}\left(\omega \right)}-1\right], \end{array}$$

where $g_{A}\left(\omega \right)$ and $g_{B}\left(\omega \right)$ are defined as in Eq. **3**. To calculate A’s distance to criticality, we numerically minimize J(A||B) over all critical AR models B of the same order as A (Fig. 2*F* and *SI Appendix*, section S4.4). We can also calculate A’s distance to each type of criticality that AR models exhibit; specifically, for each β, we define $d_{\beta }$ as the minimum of J(A||B) over B in the set of critical AR models of type β. The nested structure of the tRG basins guarantees that $d_{2}\leq d_{4}\leq d_{6}\leq \cdots$.

For example, a random walk is an AR(1) with $\phi_{1}=1$, which places it on the β=2 basin of attraction; it has $d_{2}=0$. In contrast an AR(1) with $\phi_{1}=0.99$ is close to criticality—has small $d_{2}$ - but is in the trivial β=0 basin. For model order n>1, the set of models in the β=2 basin is much more diverse than the random walk, but all these models share the same low frequency behavior.

With this practical implementation of our definition of proximity to criticality, we arrive at a two-step pipeline for estimating proximity to criticality for real data (Fig. 2*G*) (77). Step 1: fit an AR model to the real data. Step 2: use our tRG results and formula for KL divergence rate to measure the distance from the best fit model to the nearest model on the critical manifold.

## 2. Benchmarking on Other Time Series Models

**Fig. 3.**
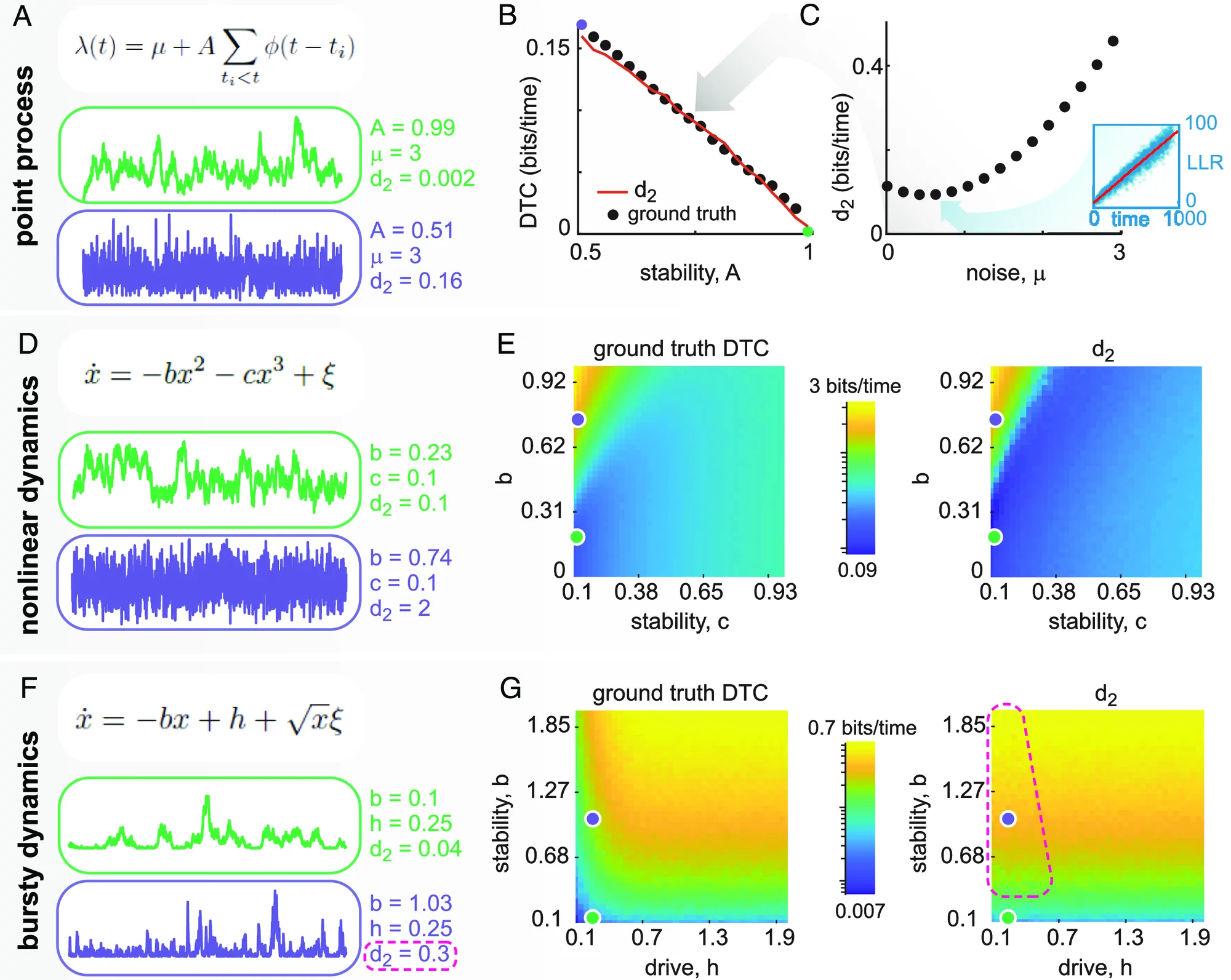
Benchmarking $d_{2}$ against ground truth cases. (*A*) A Hawkes process is at criticality for A=1, for all μ. Example “spike” count time series near criticality (green) and far from criticality (purple). For each parameter set, we simulated $10^{6}$ spikes. (*B*) $d_{2}$ closely matches ground truth DTC across a wide swath of parameter space, including models far from criticality (A≪1) and models close to criticality (A∼1). (*C*) The ground-truth DTC values in (*B*) were calculated by minimizing the KL divergence rate over the set of critical Hawkes processes. *Inset*: numerical calculation of KL divergence rate. (*D*) Langevin dynamics in a quartic potential is at criticality for b=c=0. We simulated $10^{6}$ time steps for each parameter set. (*E*) Ground truth DTC agrees well with $d_{2}$ as a function of b and c (correlated with Pearson $r^{2}=0.95$). (*F*) The bursty model is at criticality for b=0, for all h. We simulated $10^{6}$ time steps for each parameter set. (*G*) $d_{2}$ matches ground truth DTC well (correlated with Pearson $r^{2}=0.79$). When h is small (red dashed line), $d_{2}$ does not match ground truth, which is when the model dynamics deviate extremely from Gaussian, precluding a good AR model fit.

**Fig. 4.**
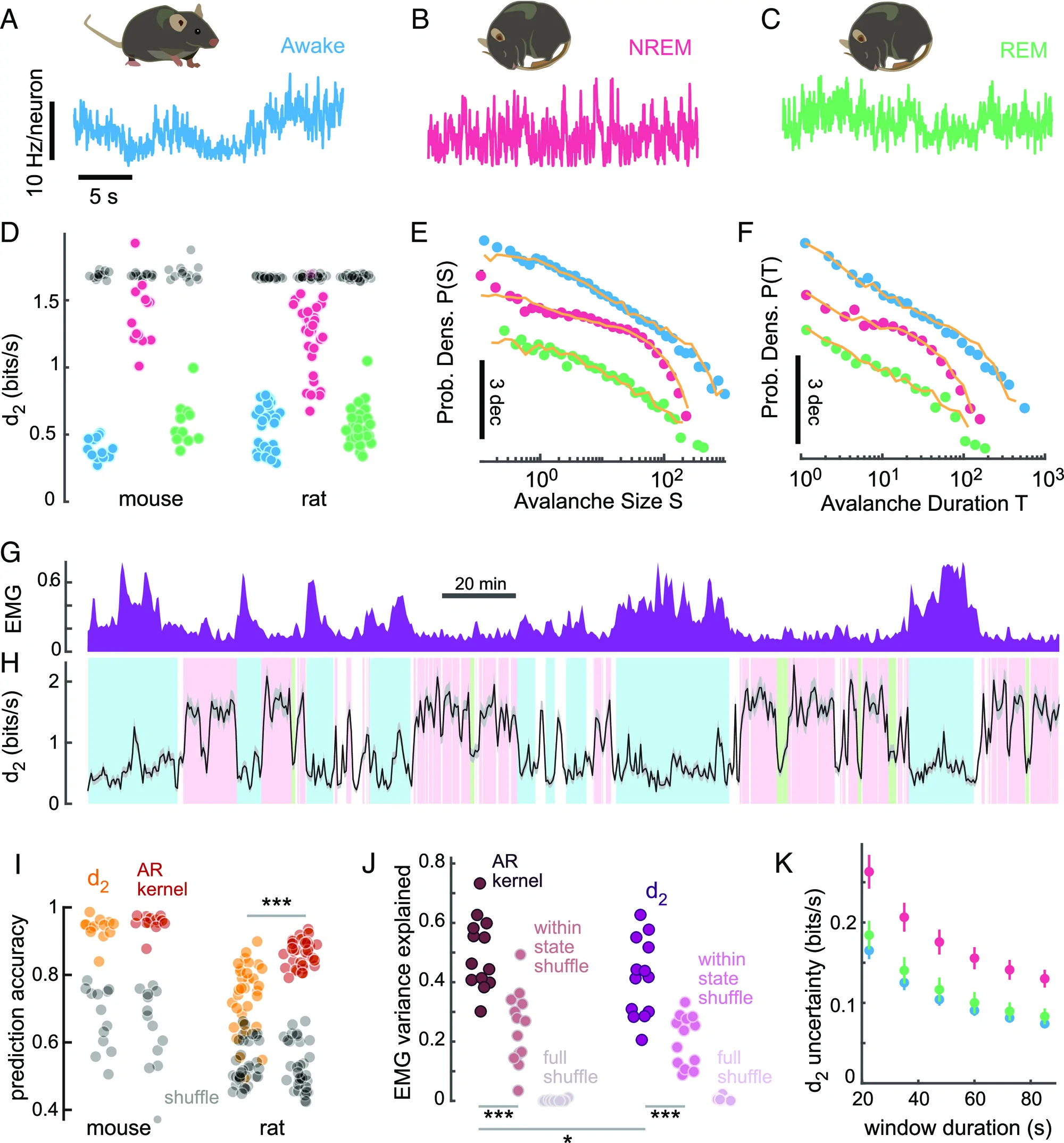
Estimated proximity to criticality in mammalian brain depends on vigilance state and behavior. (*A*–*C*) Mouse V1 activity during wakefulness, NREM sleep, and REM sleep. Each row of dots represents spike times from a single neuron. We apply our approach to spike count time series (colored lines). (*D*) V1 is closest to criticality ($d_{2}$) during wake (blue) and furthest during NREM (red). Faded gray dots are for rate-matched Poisson surrogate data. Each point represents a single recording session. (*E*) Avalanche size distributions for mouse V1 during each behavioral state (markers) match well to avalanche size distributions from simulations of best-fit AR models (gold lines). These examples are from the same recording session as shown in (*A*–*C*). The exponent of the best fit power-law in the awake state was 1.50 ± 0.03 (size) and 1.56 ± 0.07 (duration) (mean ± s.e.m. across sessions). (*F*) Same as (*E*), but for avalanche duration. (*G*) Body activity (electromyography, EMG), obtained simultaneously with the $d_{2}$ timeseries in (*H*). (*H*) $d_{2}$ calculated in a 60 s sliding window reveals time-resolved shifts in proximity to criticality (black line). Gray shaded area represents uncertainty $\Delta d_{2}$. Color of background indicates wake (blue), NREM (red), or REM (green) periods. (*I*) Sleep/wake states can be predicted with high accuracy using $d_{2}$ (orange) and even higher accuracy using all AR model coefficients (red). (*J*) Body movement (EMG) can be predicted using $d_{2}$ nearly as well as prediction based all AR model coefficients (*P<0.05). EMG fluctuations within each sleep/wake state are predicted by $d_{2}$ (***P<0.001), as evidenced by drop in prediction accuracy when EMG is shuffled within each state. (*K*) Uncertainty in $d_{2}$ depends on sleep state and decreases for increasing duration of the sliding window duration.

While AR models are justified in the *max ent* sense, it is not immediately obvious that AR models are a good fit for brain data. The brain is nonlinear and multivariate, population activity is sometimes highly non-Gaussian, and many important experimental measurements are of spikes. In contrast, AR models are linear, Gaussian, and do not directly model spikes. Can our approach accurately measure proximity to criticality using data that were not generated by an AR model? Next we show that our tools work surprisingly well in four cases: 1) a point process model that generates spike-like data (Hawkes process, Fig. 3*A*–*C*), 2) a nonlinear time series model (overdamped Langevin equation, Fig. 3 *D* and *E*), 3) a non-Gaussian bursty model (Fig. 3 *F* and *G*), and 4) a nonlinear network model of 1000 binary, spiking neurons (*SI Appendix*, section S3). For these models, critical parameter configurations are known. We compared $d_{2}$ to the ground truth DTC for each model, which we obtained by analytical and numerical methods (*SI Appendix*, sections S5.1–S5.4). True DTC was faithfully revealed by $d_{2}$ in all cases except for very bursty dynamics (red dashed area Fig. 3*G*). We conclude that our AR-based approach work well for our goals, but not without limitations.

## 3. Application to Cortical Spiking Activity

As a first use case for our approach, we asked whether the mammalian brain is closer to criticality during sleep or during wake. Previous studies report inconsistent results; some suggest sleep is closer to criticality than wake (78), others suggest the opposite (24, 60), and some report little difference (30, 79–81). Here, we performed electrophysiological recordings of single-unit spike activity from primary visual cortex of freely behaving rats (first reported in ref. 30, 77, 90)) and mice (first reported in ref. 82). We analyzed 96 to 240 h of data recorded in each of N=8 rats, with a mean of 35 ± 7.9 single units per 12 h clustering block (see ref. 30 for details). For the mice, we analyzed 13 recordings, 6.2±1.8 h in duration (mean ± sd), 67±18 neurons per recording. In both datasets, periods when the animals were awake, in deep sleep (NREM sleep), or in REM sleep were determined based on animal movement and local field potential measurements as detailed in the original papers (30, 82). We note that these assessments of sleep/wake states did not account for the possibility of local sleep in awake animals (83) nor local desynchronization in asleep animals (72), which could contribute to some of the variability in our results.

We estimated proximity to criticality ($d_{2}$) during different states of sleep and wake. First, we created a spike count time series (Δt=40ms time bins). We chose to analyze spike count time series because this is common in previous studies of criticality based on avalanche analyses, but we note that alternative approaches for extracting a univariate time series from multivariate data might also be used (23, 66, 67). We fit an AR model to the spike count time series separately for awake (Fig. 4*A*), NREM sleep (Fig. 4*B*), and REM sleep periods (Fig. 4*C*) (*Materials and Methods*). For both mice and rats, $d_{2}$ indicated that cortical neurons were closest to criticality ($d_{2}$ was smallest) in the awake state and furthest from criticality during NREM sleep (Fig. 4*D*). Proximity to other tRG fixed points, $d_{\beta }$’s with β=4,6,.., also distinguish the sleep and wake states (*SI Appendix*, Fig. S4). For more details on how $d_{2}$ depends on choice of time bin and model order, see *SI Appendix*, Fig. S8.

For more direct comparison to previous studies, we also performed avalanche analysis (*Materials and Methods*). We created distributions of avalanche sizes and durations and used them for two purposes. First, in agreement with minimal $d_{2}$ in the wake state, we found that avalanches had the largest range of power-law scaling in the awake state (Fig. 4 *E* and *F* and *SI Appendix*, Figs. S5–S7 for exhaustive results). Second, we used avalanche analysis as an additional check of how well the fitted AR models matched the observed dynamics. Considering that real brains are nonlinear and high-dimensional, it is important to verify that a one-dimensional AR model can reproduce the complex statistics of the measured spike rate time series. We found that the AR model fit was surprisingly good. We performed avalanche analysis on time series simulated using the best fit AR model and found close agreement between the avalanche distributions of the experimental data and those obtained from the AR simulations (gold lines in Fig. 4 *E* and *F*).

These results summarize multihour recordings. Can our approach track changes in proximity to criticality on shorter time scales? We performed $d_{\beta }$ measurements in a sliding 60 s window and found that $d_{2}$ fluctuations reflected changes in sleep/wake states and body movement (EMG) (Fig. 4 *G* and *H*). The uncertainty in $d_{2}$ (*SI Appendix*, section S4.5) is small compared to $d_{2}$ changes across sleep/wake states for 60 s or even shorter sliding window durations (Fig. 4 *I* and *K*). We could predict sleep/wake states with 94±1% and 74±2% accuracy in the mouse and rat data, respectively (mean ± sem across sessions, *Materials and Methods*). More impressive, we were able to predict EMG using $d_{2}$, including EMG fluctuations within each sleep/wake state (Fig. 4*J*). Finally, we performed a benchmarking study, using $d_{2}$ to track state changes in simulated time series, in which the ground truth about state changes is known (*SI Appendix*, Fig. S1). We found that $d_{2}$ outperforms avalanche-based metrics and tracks changes accurately as long as the sliding window duration is not too short (greater than about 30 s) and is less than the period of typical state changes. Overall, these results suggest that $d_{2}$ can track proximity to criticality, even in systems with changing underlying control parameters, like the brain.

## 4. Discussion

We have proposed a formal definition of distance to criticality for univariate time series models and implemented it by developing a temporal RG and KL divergence rate for AR(n) models. AR models are used to represent time series data across a wide range of data science domains (1, 2). Our results offer a principled way to ask about proximity to criticality across these domains. Here, we leveraged our approach to address a long-standing controversy, providing strong evidence that the cortex is closer to criticality during wake than NREM sleep in two independently collected datasets from two different species (mice and rats).

For a given time series, the repertoire of timescales present in the fluctuations depends strongly on proximity to criticality. Thus, $d_{2}$ depends strongly on the timescale repertoire. How does $d_{2}$ compare to other assessments of the timescale repertoire? These include power spectra (84, 85), dominant timescale estimates (24, 49, 60), detrended fluctuation analysis (DFA) (57, 58), avalanche analysis (21, 23) and more (48). A comprehensive quantitative comparison will require further work, but we mention a few important differences. First, avalanche analysis focuses on statistics of large fluctuations—the avalanches—and is blind to what is happening between avalanches—the “quiet times.” In contrast $d_{2}$ accounts for the entire timeseries, thus accounting for temporal scale invariance differently than avalanche analysis. This could explain why some avalanche-based analyses did not find differences between wake and NREM sleep (30), while $d_{2}$ reveals a difference (Fig. 4 *D* and *H*). An advantage over avalanche analysis is that $d_{2}$ works on short time series, affording a time-resolved, sliding-window assessment of proximity to criticality (Fig. 4*G* and *SI Appendix*, Figs. S1 and S2). Comparing to methods that probe the dominant timescale of a time series (24, 49, 60), a decrease in $d_{2}$ is likely when the dominant timescale increases, as expected for “critical slowing down.” However, distance to criticality is not determined solely by the longest timescales of the system; for a fixed longest timescale, $d_{2}$ can vary over an order of magnitude (*SI Appendix*, Fig. S9). Comparing to power spectra and DFA, we note that their power-law exponents are related to criticality, but do not measure proximity to criticality. This is particularly true when the shape of the power spectrum or fluctuation function deviates from a power-law, which is expected for a system that departs from criticality.

What are the limitations of our approach and what are some next steps for extending it? A fundamental limitation of our approach is that it only applies to one-dimensional time series data. Thus, our approach cannot assess spatial aspects of scale invariance as done in some previous studies of criticality based on fMRI (70) and wide-field voltage imaging (44, 71, 72). Nonetheless, multivariate models and data often are decomposed into multiple one-dimensional modes either analytically or using empirical dimensionality reduction methods. Our approach can be applied successfully to such 1-dimensional time series (67) like the population average as we did here (Fig. 4 and *SI Appendix*, Fig. S3). Another limitation highlighted in Fig. 3 is that our approach should not be used with very bursty data. In this case, an AR model is simply a bad fit and $d_{2}$ tends to overestimate the ground truth distance to criticality. An ambitious solution would be to develop tRG and KL divergence for a different class of models that can fit bursty data (replacing Section 1.3 of our work). If this could be done, then one could swap these new results for our AR model results and proceed with the rest of our pipeline. Another limitation of our conclusions is that we have applied our approach only on spike data. It is natural to ask whether our approach works for brain activity time series measured with other methods—for instance, local field potential (LFP), electroencephalography (EEG), or functional MRI (fMRI). If these signals can be well-described by an AR model, then our approach will work. However, some caution is appropriate when comparing $d_{2}$ based on spikes to $d_{2}$ based on other collective signals. For instance, LFP is typically band-pass filtered (1 to 100 Hz), which cuts out many of the long time scale fluctuations that are prominent in awake spike data (e.g. Fig. 4*A*) and play an important role in our theory. Understanding how to reconcile our methods across different types of measurement modalities will require future work.

There are many open questions about proximity to criticality and brain function. How do deviations from criticality relate to brain disorders (26, 33, 86, 87)? Does distance to criticality vary across brain regions (71, 88)? Do moment-to-moment shifts in proximity to criticality impact cognitive performance (24, 34) ? We have established a solid theoretical foundation for these questions and theory-driven data analytic tools (and freely available code: https://github.com/sam-sooter/prox_crit_toolkit) to answer them using measured univariate time series data.

## 5. Materials and Methods

### 5.1. Rat Experiments.

Rats were implanted with an 64-electrode array (16 tetrodes made of 12 μm wires) in the primary visual cortex under isoflurane anesthesia as detailed previously (30). Implantation coordinates were 1.45 mm/3.45 mm/ −1 mm (anteroposterior/mediolateral/dorsoventral relative to lambda and dura). The total headstage weight, including cement, bone screw, and enclosure, was 2 g. After 2 d of recovery, neural activity was recorded (25 kS/s, eCube Server, White Matter) continuously for 10 to 14 d during free behavior in home cage. Animal behavior was monitored with 15 or 30 fps video recording synchronized to the electrophysiological data with Watchtower software (White Matter). Raw electrophysiological data were bandpass filtered between 500 and 7,500 Hz and thresholded for spike-waveform extraction (mean ± 4 s.d.). Spike sorting was performed with a modified version of SpikeInterface63 and MountainSort4 as described in ref. 30.

### 5.2. Mouse Experiments.

As reported previously (82), mice were implanted with a 64-channel silicon probe (single shank, Cambridge NeuroTech, H3 64 × 1 probe) mounted on a movable microdrive for recording the activity of multiple single-units and local field potentials (LFPs) in the primary visual cortex. The probe was implanted at 1.0 mm/2.5 mm anteroposterior/mediolateral, with a 21 degree angle from dorso-ventral axis and a 10 degree angle from the mediolateral axis. The probe tip was 1.0 mm deep after insertion. After overnight recovery in their home cage, extracellular electrophysiological recording were performed while the animals slept or walked around freely moving in the home cage for 68 h. Electrophysiological data were acquired using an Intan RHD2000 system (Intan Technologies LLC) sampled at 20 kHz rate. Spike sorting was performed semiautomatically, using Kilosort.

### 5.3. Calculating $d_{2}$ from Segmented Time Series.

In Fig. 4*D*, we reported a single $d_{2}$ value for each state (wake, NREM, or REM). To obtain a $d_{2}$ for NREM, for instance, we first calculated the autocorrelation function separately for each NREM segment and then averaged across all segments (accounting for variable lengths of segments). Then we used the Yule–Walker method to obtain best-fit AR model coefficients from the average autocorrelation function. We then used these coefficients to calculate $d_{2}$. In contrast, in the time-resolved $d_{2}$ analysis in Fig. 4*H*, we used maximum likelihood estimation (MLE) of the AR model coefficients because this allowed us to estimate error bars for $d_{2}$ (*SI Appendix*, section S4.5).

### 5.4. Avalanche Analysis.

To generate the avalanche distributions in Fig. 4 *E* and *F* and *SI Appendix*, Figs. S5–S7, we used standard procedures, as described elsewhere (e.g., ref. 23). In brief, the first step is to create a spike count time series $x_{1},x_{2},...$, where $x_{i}$ is the total number of spikes from all recorded neurons in the ith time bin. Time bins are nonoverlapping, consecutive, and 40 ms in duration. Then, avalanches are defined as periods of time when the spike count exceeds a threshold (10th quantile). For each avalanche, its size is defined as the area between the threshold and the spike count time series and its duration is defined as the time spent above the threshold. Each size or duration probability density distribution was created using 10 logarithmically spaced bins per decade. Each recording had 1,000s of avalanches. However, avalanche analysis is not feasible in short time windows like the sliding window $d_{2}$ analysis in Fig. 4*H*, because there are not enough avalanches in such a short period to obtain an accurate distribution. We further detail how $d_{2}$ works better with such short time series in *SI Appendix*, Fig. S1.

### 5.5. Predicting Sleep States from $d_{2}$.

We used a multinomial logistic regression (Matlab’s mnrfit function) to predict sleep state from $d_{2}$ and the AR history kernel (Fig. 4*I*). The model linearly combines the predictor variables (either $d_{2}$ or the kernel) to compute a score for each class (NREM, REM, wake), converts these scores into class probabilities using the softmax function, and outputs the class with the highest probability. We evaluated model performance with fivefold cross-validated classification accuracy.

### 5.6. Predicting EMG, Gradient-Boosted Decision Trees.

We used gradient-boosted decision trees (XGBoost algorithm) to predict body movement (electromyogram, EMG; Fig. 4*J*) from $d_{2}$ and the AR history kernel. To select hyperparameters without data leakage, we used nested, temporally contiguous cross-validation. Specifically, we first split each session into temporal halves (“outer” cross-validation folds). We then split each half into two further temporal halves (“inner” folds). Next, we used Bayesian optimization (BayesSearchCV function from scikit-optimize package) to select hyperparameters that maximized cross-validated performance (measured as the mean squared prediction error) over the inner folds. Finally, we reported the model performance as the cross-validated performance across the outer folds. We selected hyperparameters from the following ranges: “learning_rate” (0.01, 0.3), “n_estimators” (50, 500), “max_depth” (3, 10), “subsample” (0.5, 1), “colsample_bytree” (0.5, 1), “reg_alpha” (0, 10), and “reg_lambda” (0, 10).

## Supplementary Material

Appendix 01 (PDF)

## Data Availability

Code for calculating d is freely available on GitHub (89). The mouse data is available for download from the Buzsaki Lab data repository (77) and from Figshare (90). We analyzed 13 datasets with the following labels in the Buzsaki Labdatabase: YMV02, YMV07, YMV08, YMV09, YMV10, YMV11, YMV12, YMV13, YMV14, YMV15, YMV16, YMV17, and YMV18. The raw datasets for the rat data constitute >10 terabytes of raw neural broadband. The data are stored in a cost-efficient manner, but not immediately available for download. Data are available upon request (khengen@wustl.edu).
