# Supplementary material for: Defining and measuring proximity to criticality based on univariate time series
Source: Proc Natl Acad Sci U S A. 2026 Oct 28;123(44):e2526984123. doi: 10.1073/pnas.2526984123 (PMC13732201; doi:10.1073/pnas.2526984123)
Supplement: Supplementary file 1 [file pnas.2526984123.sapp.pdf]

# SUPPLEMENTARY MATERIALS FOR "DEFINING AND MEASURING PROXIMITY TO CRITICALITY"

J. SAMUEL SOOTER, ANTONIO J. FONTENELE, ANDREA K. BARREIRO, CHENG LY, KEITH B. HENGEN, AND WOODROW L. SHEW

Contents


1. Time resolution of $d_2$ analysis 2
1.1. Tracking state changes with sliding window analysis 2
1.2. Bias and uncertainty in $d_2$ for short time series 2
2. Additional test of $d_2$ in multivariate spiking model 5
2.1. Model details. 6
2.2. Simulations and analysis of the model dynamics. 6
3. Additional details of mouse and rat spike data analysis 7
3.1. Distances to other fixed points - $\beta > 2$. 7
3.2. Avalanche distributions. 7
4. Distance to Criticality for AR Models 7
4.1. Probability Distribution of Trajectories 7
4.2. tRG Basins of Attraction 11
4.3. KL Divergence Rate for AR models 13
4.4. Numerical Minimization of KL Divergence Rate 14
4.5. Further details on AR model estimation and $d_2$ error bars 15
5. Model Benchmarking 16
5.1. Numerical calculation of KL divergence rate 16
5.2. Univariate Hawkes Process 17
5.3. Nonlinear model: Langevin dynamics in quartic potential 18
5.4. Bursty model: Langevin dynamics with multiplicative noise 19
6. Dependence on Time Bin Length and AR Model Order 20
7. Relating $d_2$ to the longest time scale of the dynamics 20
8. Restricting the set of critical models to its intersection with a tractable family 21
References 23

## 1. Time resolution of $d_2$ analysis

In Fig. 4H of the main text, we performed sliding window measurements of $d_2$, revealing behaviorally-related changes in estimated proximity to criticailty. Moreover, we showed that error bars for these measurements (Supp. Mat Section 4.5) were small compared to differences in $d_2$ across different behavioral states for sliding windows as short as about 20 s (Fig. 4K). This time resolution is better than that of avalanche-based measures of proximity to criticality. This is one of the major advantages of our approach. Here we take a closer look at the limits of this time resolution.

1.1. **Tracking state changes with sliding window analysis.** First we consider a test case in which we control when state changes occur. We measure the accuracy with which we can track these state changes. We show that $d_2$ outperforms power-law range (for both avalanche size and duration distributions) and the deviation from criticality coefficient (DCC). Power-law range is defined by fitting a truncated power-law to an avalanche distribution and seeking the largest range that meets a given goodness-of-fit criterion [1, 2]. According to theory, power-law range should be larger for a system that is closer to criticality (e.g. Ref [3]). DCC has been used to measure deviation from criticality based on how well the avalanche size and duration distributions conform to the crackling noise scaling law [4, 5, 6, 7]. Here we show in Fig. S1 that, when limited to short duration time series, as in sliding window analyses, $d_2$ outperforms power-law range and DCC.

We simulated time series using AR(5) models with known history kernels and, thus, known ground truth distance to criticality. Each simulation switched between three different states (i.e. three history kernels). The three states were obtained by fitting an AR(5) to experimental data, one based on NREM sleep periods, another based on quiet wake (defined as waking periods with EMG below its $25^{th}$ percentile), and the third based on active wake (defined as waking periods with EMG above its $75^{th}$ percentile). Thus, our test cases are comparable to the state changes we observed in the real data, but we have precise ground truth knowledge of the state changes that our $d_2$ analysis should reveal. Comparing the estimated $d_2$ to the ground truth $d_2$ affords quantitative assessment of how well our approach works. As shown in Fig. S1, our approach works very well (above 80% accuracy) as long as the window duration used for sliding window analysis is not too short (greater than about 30 s) and remains smaller than the typical durations of the ground truth state changes.

We also performed avalanche analysis on the simulated time series using the same analysis parameters as in the main text (40 ms time bin, 10 percentile avalanche threshold). Power-law range and DCC perform dramatically worse when subjected to the same quantitative tests (Fig. S1B,C). This poor performance is due to the fact that avalanche analysis, in general, requires a large number of avalanches to obtain a reasonable estimate of each avalanche distribution, which in turn requires long recordings. In other words, the accuracies of avalanche-based measures are fundamentally limited by the simple fact that a probability distribution will be noisy if there are not enough samples.

1.2. **Bias and uncertainty in $d_2$ for short time series.** Next we quantify how error in $d_2$ depends on the duration of the analyzed time series (Fig. S2). Most importantly, we found that for time series with more than a few hundred samples (like those we studied in Fig. 4 in the main text), the bias and uncertainty is very small. We simulated an AR(1) model for different durations between 10 and $10^4$ time steps. We did this for a range of states from $\phi = 0$ to $\phi = 1$, where $\phi$ the kernel coefficient. We performed $10^4$ simulations for each choice of $\phi$ and time series duration. For each simulation we estimated $d_2$ with our tools and compared to the ground truth distance to

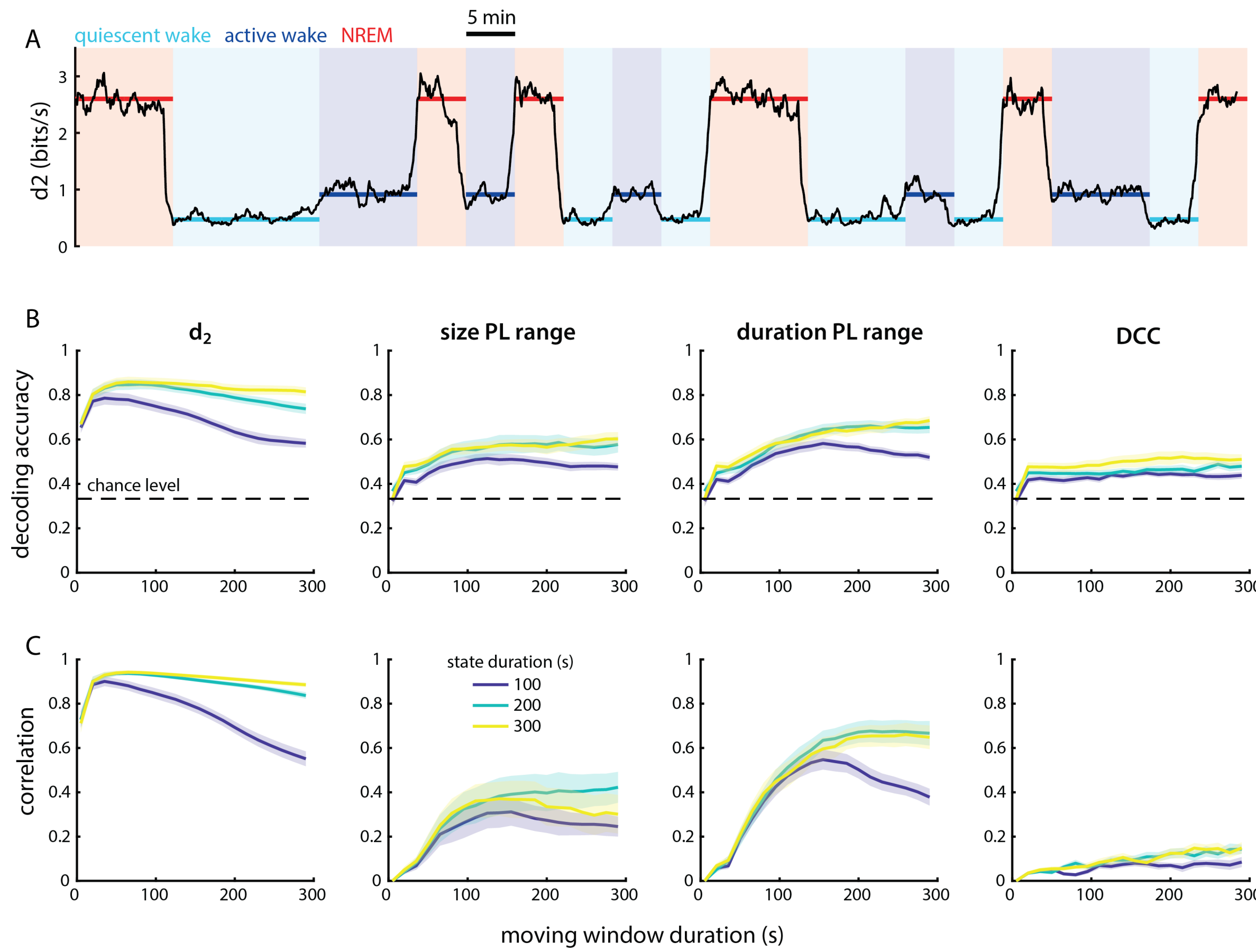


FIGURE S1. $d_2$ **tracks changing proximity to criticality better than other metrics.** **A)** We simulated an AR(5) that randomly switches among 3 different history kernels. Each kernel was obtained by fitting an AR(5) model to experimental data: one kernel was fit to NREM sleep data (red), another was fit to quiescent wake (defined as EMG below the first quartile of wake state EMG, cyan), and the third was fit to active wake (defined as EMG above the third quartile of wake state EMG, blue). The black line shows $d_2$ calculated in a sliding window (60 s window duration, 5 s slide step). Note that $d_2$ correctly tracks the changing state, but not perfectly. **B)** We quantified how well $d_2$ tracks state changes by training a classifier (10-fold cross-validated logistic regression) to decode the true underlying state from $d_2$. We varied the switching period of the underlying state (100 s - yellow, 200 s - cyan, 300 s - purple) and the sliding window duration from 5 to 300 s. As long as the sliding window was not too short (more than about 30 s) and remained less than the switching period, $d_2$ surpasses 80% accuracy. Other metrics including power-law range of avalanche sizes (second column) and durations (third column) and the deviation from criticality coefficient (DCC) were less accurate. The line and shaded areas represent mean $\pm$ s.e.m. over multiple realizations with different ground truth states obtained by fitting to different experiments (all the mouse data sets presented in Fig. 4 of the main text). **C)** Same as panel B, but quantifying correlation between ground truth $d_2$ and our short time estimate of $d_2$. Similar to decoding accuracy, the correlation between $d_2$ and ground truth state changes is greater than that for other metrics.

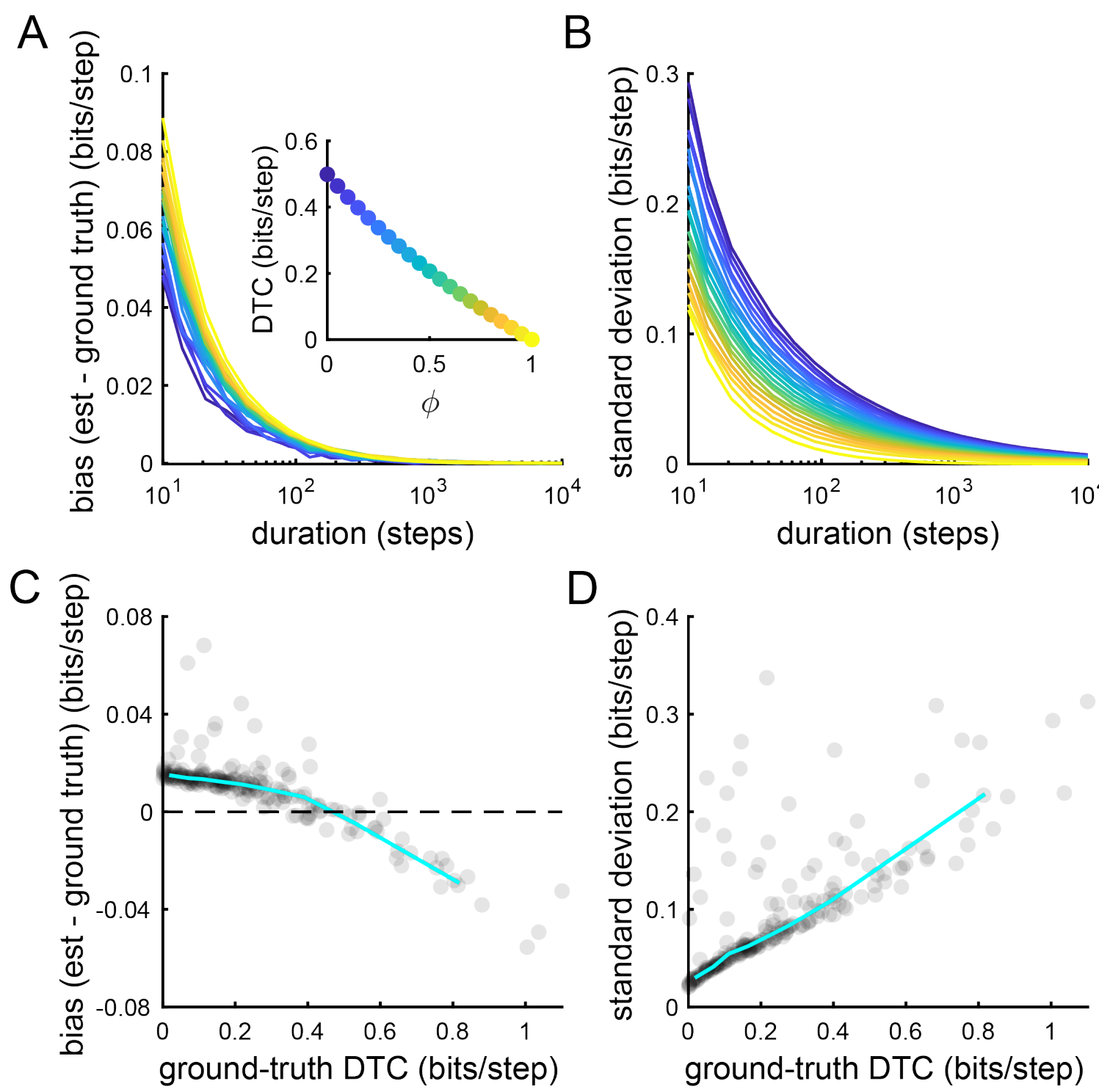


FIGURE S2. **Estimated $d_2$ slightly over estimates true $d_2$ for short time series.** **A)** Estimated $d_2$ is slightly above the true $d_2$ for AR(1) models; this over estimation increases for shorter time series. Inset shows ground truth $d_2$ and model coefficient $\phi$ for each color. **b)** Variability in estimated $d_2$ also increases for shorter time series. Variability is less for models near criticality. **c)** Each point represents a the bias for one randomly chosen AR(3) model simulated for 50 time steps. Bias tends to be positive except for cases very far from criticality. **d)** Varibility for the same AR(3) models shown in panel (C).

criticality (KL divergence rate). We define 'bias' as the difference between our estimated $d_2$ and the ground truth $d_2$, averaged over the $10^4$ realizations. To quantify uncertainty, we also measured the standard deviation across the $10^4$ realizations.

We found that the bias is positive, meaning that estimated $d_2$ overestimates the ground-truth; the bias is positive and decreases towards zero as the time series duration increases. Moreover, the tendency to overestimate is most severe when the ground-truth state is close to criticality. This makes sense intuitively - it should be hard to tell the difference between, say, a model with a 5 s autocorrelation time and one with a 10 s autocorrelation time if we only look at 1 s snippet of activity. The bias and uncertainty both go to zero as the duration increases. Also, the uncertainty is higher for models that are farther from criticality (Fig. S2A,B).

Next, we asked whether the qualitative relationships between ground-truth DTC and bias/uncertainty observed for AR(1) models hold more generally. Specifically, we randomly selected many (250) different AR(3) models and calculated the bias and uncertainty of the d2 using a simulation duration of 50 time steps. In Fig. S2C,D, each point corresponds to one model and the cyan lines show a moving median of the points. Similar to AR(1) models, the models closest to criticality have

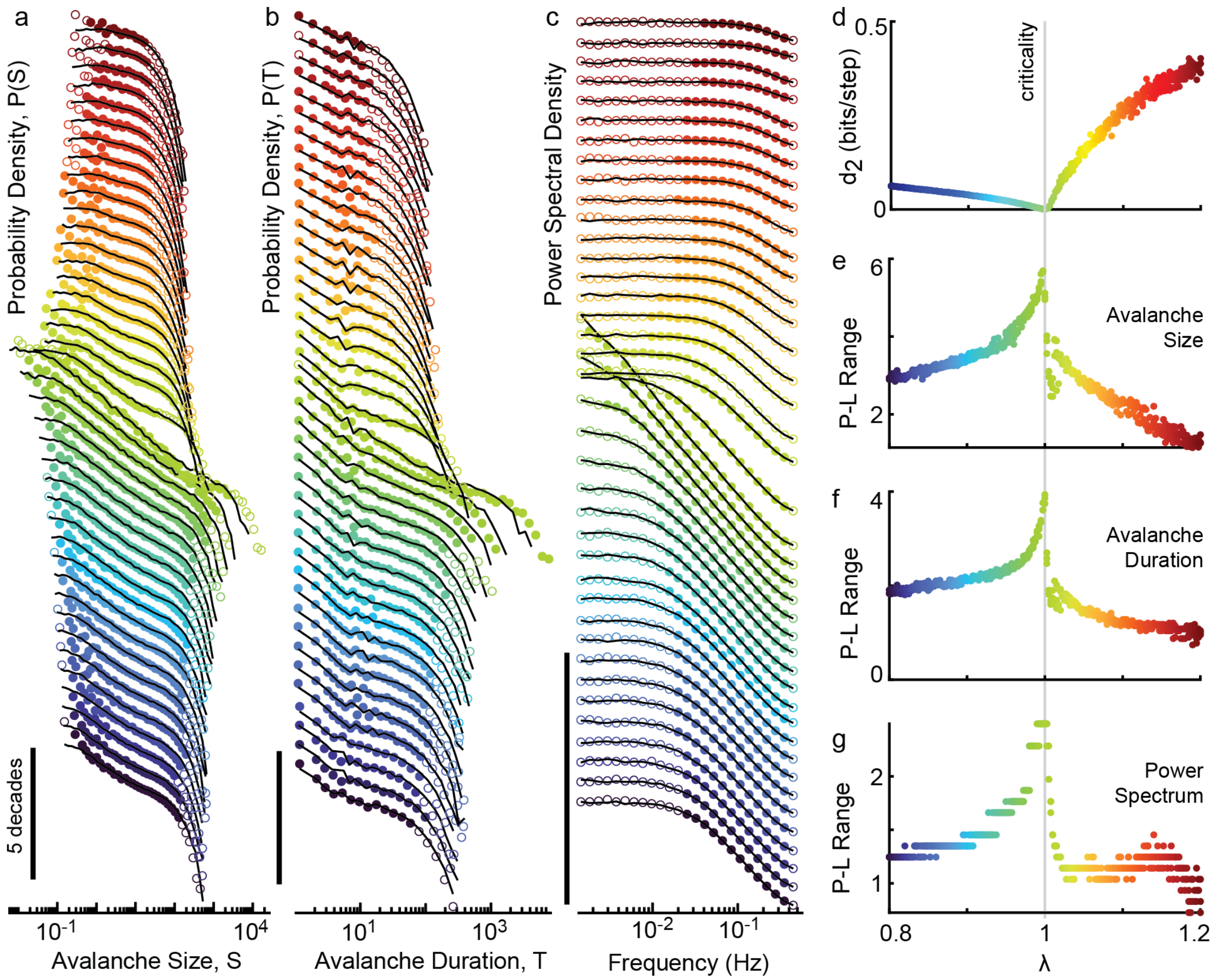


Figure S3. $d_2$ **tracks distance from criticality in multivariate model. a)** Avalanche size distributions for a family of models ranging from subcritical (blue, $\Lambda = 0.8$) to critical (green, $\Lambda = 1.0$), to supercritical (red, $\Lambda = 1.2$). Black lines were obtained from simulations of the best-fit AR model for each value of $\Lambda$. Filled circles indicate the portion of the distribution that is statistically well-fit by a power-law. **b)** Same as panel (a), but for avalanche durations. **c)** Same as panel (a), but for power spectra. **d)** $d_2$ smoothly goes to zero as $\Lambda$ approaches 1, thus reflecting distance to criticality. **e-g)** Power-law range gradually decreases as $\Lambda$ departs from 1. Note, however, that power-law range estimates are messy near criticality on the supercritical side. This shows that $d_2$ provides a less noisy estimate of proximity to criticality than power-law range.

the highest positive (overestimation) bias and the uncertainty is higher for models farther from criticality.

## 2. Additional test of $d_2$ in multivariate spiking model

In Fig. 3 of the main text, we present three benchmarking studies, showing that our estimate of proximity to criticality $d_2$ matches the ground truth distance to criticality for diverse models (except for very bursty dynamics, Fig. 3G). This accuracy is surprising in the sense that the test case models in Fig. 3 were fundamentally different than the AR model our approach is based on.

We successfully assessed proximity to criticality for a point process and two nonlinear models, one of which had multiplicative noise. However, the application of our approach to brain data, or other multivariate systems, raises the question: can $d_2$ track proximity to criticality based on a one-dimensional time series generated by such a high-dimensional dynamical system? The general answer to this question depends on precisely how one extracts a 1-dimensional time series from the high-dimensional dynamics [8]. Here we test our $d_2$ analysis on a population spike count time series (summing spikes from all neurons in each time bin) generated by a multivariate model (Fig. S3). We find that our approach can indeed track proximity to criticality in a model comprising 1000 binary neurons (80% excitatory, 20% inhibitory). This model is also nonlinear.

2.1. **Model details.** The model we study here has been used in previous studies of criticality in neural systems [9, 10]. The model is a network of $N = 1000$ binary neurons comprising excitatory neurons (800 neurons) and inhibitory neurons (200 neurons). The state $S_i(t)$ of neuron $i$ at time step $t$ is 1 for firing, 0 for inactive. At time step $t$, neuron $i$ fires with probability $\rho_i(t)$,

$$\rho_i(t) = f\left(\sum_{j=1}^{N} C_{ij} S_j(t-1) + P\right) \tag{1}$$

where $C_{ij}$ is the strength of the connection from neuron $j$ to neuron $i$ and the sum in the first term represents the total input to neuron $i$ from all neurons that were active at time $t-1$. The function $f(x)$ is piecewise linear: $f(x) = 0$ for $x \leq 0$, $f(x) = x$ for $0 < x < 1$, and $f(x) = 1$ for $x \geq 1$. $P$ is a time-independent, neuron-independent rate of spontaneous firing, set to $P = 0.001$.

As shown previously, our model can be tuned through a phase transition by tuning the largest eigenvalue $\Lambda$ of the connectivity matrix $C$ [1, 10, 9]. When $\Lambda$ is below 1, the model tends to generate asynchronous, low rate firing. When $\Lambda$ is above 1, the firing is also asynchronous, but saturates at a high rate. Criticality occurs at $\Lambda = 1$.

To create C, each entry in is initially set to unity with probability 0.1 and to 0 zero otherwise. 20% of the neurons are then selected at random to be inhibitory neurons and the corresponding columns of are multiplied by -1. Finally, we scaled C by a positive constant to set the magnitude of the maximum eigenvalue.

2.2. **Simulations and analysis of the model dynamics.** For each choice of $\Lambda$ in Fig. S3, we ran the model three times, for $5 \times 10^6$ time steps in each run. We then generated a one-dimensional population activity time series - the total number of spikes from all neurons at each time step. We analyzed this population activity in several ways: 1) avalanche analysis, 2) power spectra, and 3) our $d_2$ analysis. We performed $d_2$ analysis with an order 1 AR model. First, we confirmed that an AR(1) model can accurately reproduce the statistics of the population spike count time series generated by the model. This fact is demonstrated in Sup. Fig. S3a-c, in which the circular points represent results obtained directly from the simulated population activity time series and the black lines represent the same analysis performed on a simulation of the best fit AR(1) model. The precise match between the avalanche and power spectral statistics of the AR mode simulations and the original simulations indicates that an AR(1) can indeed accurately model the population activity of this multivariate model.

We found that $d_2$ smoothly approaches zero as $\Lambda$ approaches 1 (from either the sub- or super-critical side). This demonstrates that our approach works well for assessing proximity to criticality in this high-dimensional, nonlinear, spiking model. We note that a limitation of the results here is that we did not calculate a rigorous ground truth KL divergence rate distance to criticality for

this multivariate model. Rather, we assume that KL divergence rate smoothly goes to zero as $\Lambda$ approaches 1.

## 3. Additional details of mouse and rat spike data analysis

3.1. **Distances to other fixed points - $\beta > 2$.** In the main text, we reported $d_2$ measurements for the spike data in different sleep and wake conditions. We focused on $d_2$, because it is the most general distance to criticality, but our tools also measure distances to other tRG fixed point basins $\beta = 4, 6, ...$ up to $\beta = 2n$, where $n$ is model order. In Supplementary Fig. S4, we show these additional $d_\beta$ measurements. Notice that differences between the three sleep/wake states are apparent not just for $d_2$. For instance, $d_6$ seems to distinguish wake versus REM better than $d_2$. The gray rectangles indicate that our algorithm for computing distance to the nearest (min KL divergence rate) critical model did not converge for $\beta$ =16 and 18. This limitation has, in our experience, only impacted these very large $\beta$ cases.

3.2. **Avalanche distributions.** For comparison to previous studies based on avalanche analysis, we performed avalanche analysis on the mouse (Fig. S5) and rat data (Figs. S6,S7). We observe that in the awake state, the avalanche distributions tended to have a greater range of power-law scaling, which is consistent with our findings that wake state is closest to criticality, based on $d_2$. Furthermore, for each recording, we ran a simulation of the best fit AR model and performed avalanche analysis of the simulated time series. The good match between the simulated and experiment avalanche statistics verifies that AR models are good fits to our data.

## 4. Distance to Criticality for AR Models

4.1. **Probability Distribution of Trajectories.** Fix a stationary AR model with kernel $\phi = (\phi_1, \ldots, \phi_n)$ and noise standard deviation $\sigma$. Run the model for $T$ time steps to generate a trajectory $\mathbf{x} = (x_1, \ldots, x_T)$, and let $C$ be the $T \times T$ covariance matrix for this process. Then

$$P(\mathbf{x}) \propto \exp\left[-\mathbf{x}^T C^{-1} \mathbf{x}\right]$$

Since this model is stationary, $C_{tt'} = C(t - t')$. In the large $T$ limit, $C$ is diagonalized by a Fourier transform. To see this, let $F$ be the $T \times T$ matrix with entries $F_{kl} = e^{-i\omega_l k}$, where $\omega_l = -\pi + \frac{2\pi l}{T}$. Then

$$(CF)_{tl} = \sum_{k=1}^{T} C_{tk} F_{kl} = \sum_{k=1}^{T} C_{tk} e^{-i\omega_l k} = \sum_{k=1}^{T} C(t-k) e^{-i\omega_l k} = e^{-i\omega_l t} \sum_{k=1}^{T} C(t-k) e^{-i\omega_l (k-t)} \approx S(\omega_l) F_{tl} \tag{2}$$

where

$$S(\omega_l) = \sum_{t=-\infty}^{\infty} C(t) e^{-i\omega_l t}$$

is the power spectral density of the model and in the last step we assumed that $C(t)$ decays to zero at large lags. In words, Eq. (2) says that Fourier modes are (approximately, for large $T$) eigenvectors of the covariance matrix with eigenvalues given by the power spectral density. But then

$$\mathbf{x}^T C^{-1} \mathbf{x} \approx \mathbf{x}^T (F \mathrm{diag}(S(\omega_1), \ldots, S(\omega_T)) F^{-1})^{-1} \mathbf{x} = \mathbf{x}^T F \mathrm{diag}(S(\omega_1)^{-1}, \ldots, S(\omega_T)^{-1}) F^{-1} \mathbf{x}$$

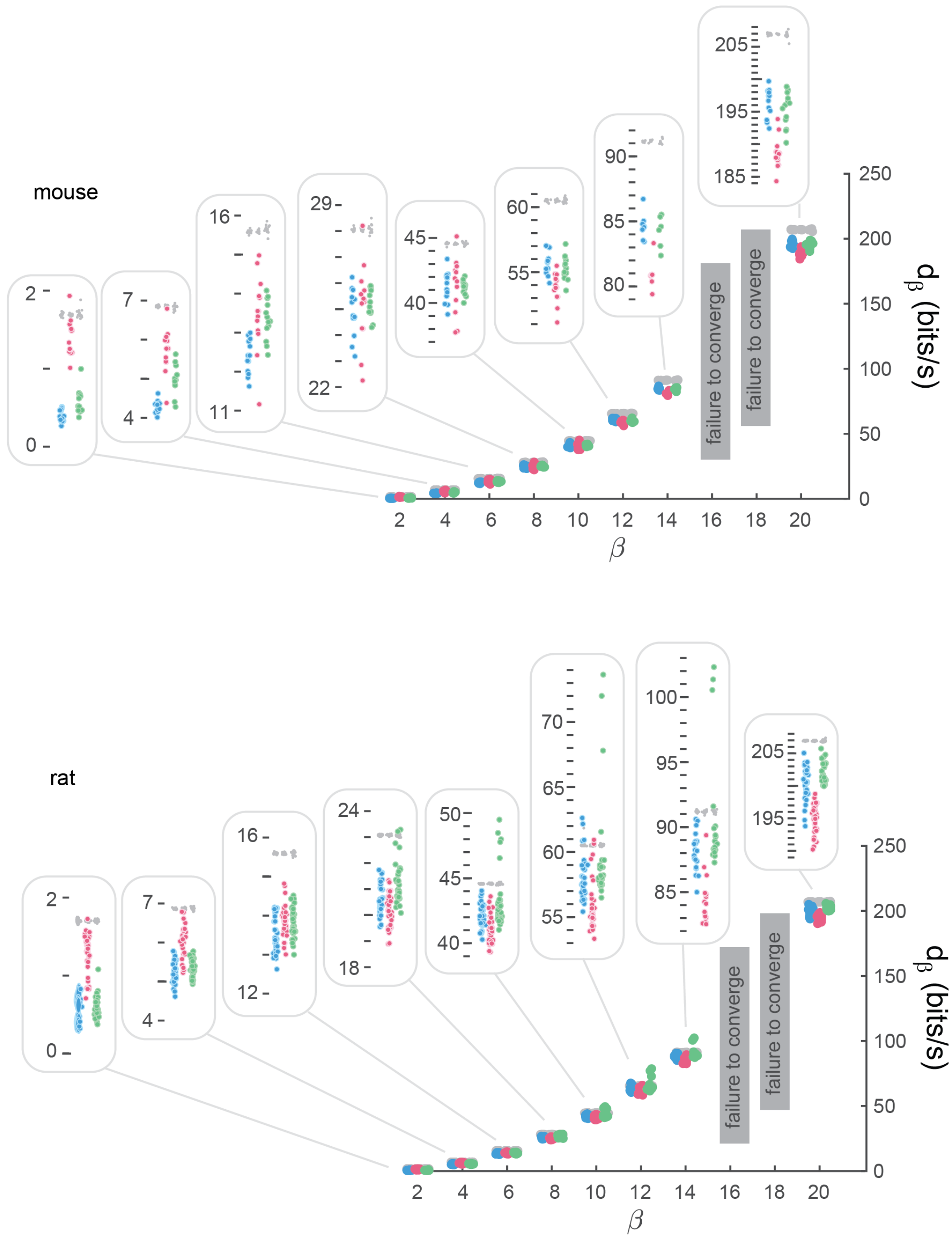


FIGURE S4. **Distances to all fixed points.** An AR(10) model was fit to each of the wake (blue), NREM (red), and REM (green) states presented in Fig. 4 of the main text. Thus, there are 10 tRG non-trivial fixed points to consider. In general, distances $d_\beta$ increase with $\beta$. Each inset zooms in on one set of $d_\beta$ results to show sleep versus wake differences.

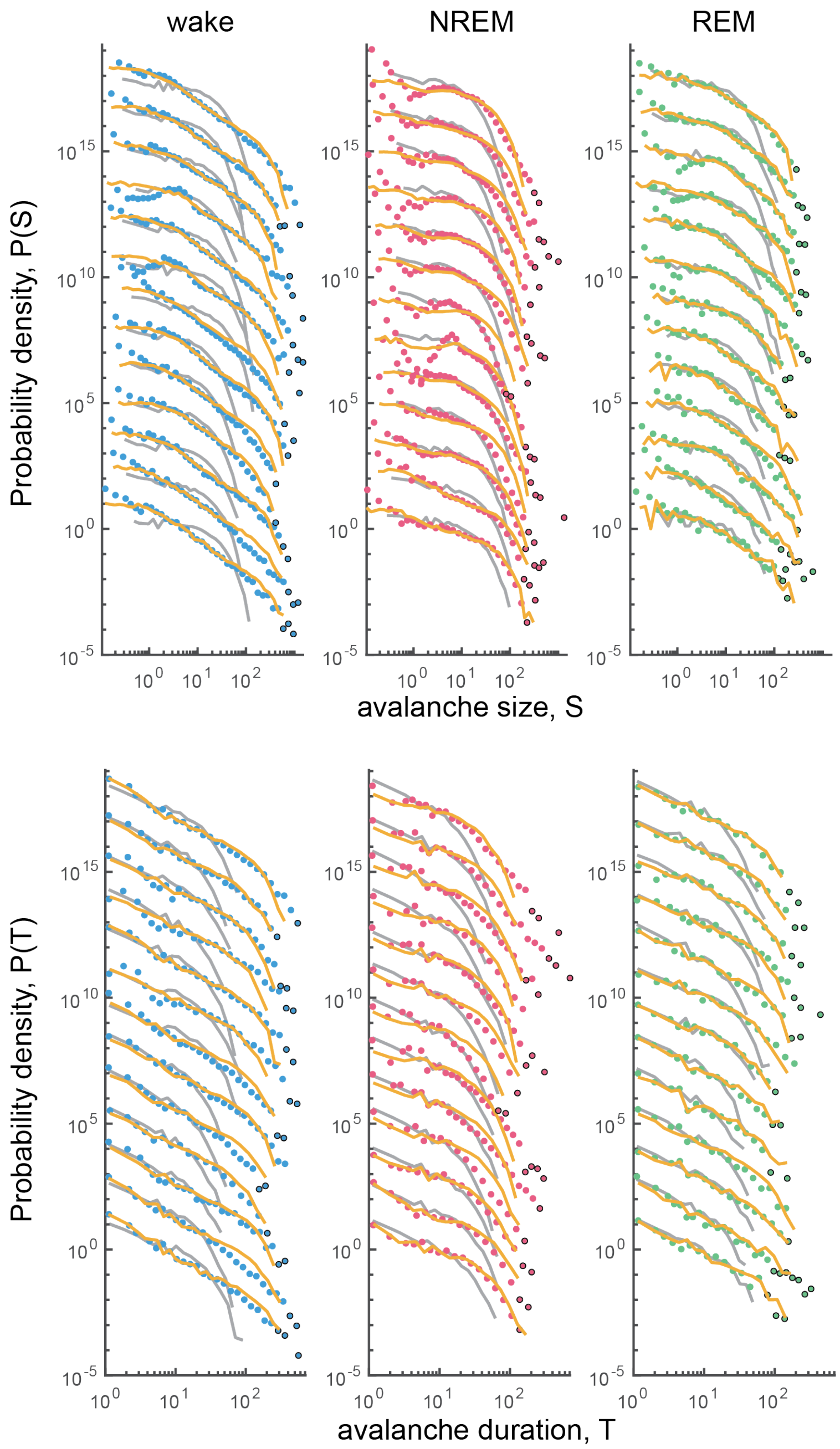


FIGURE S5. **Avalanche distributions for mouse data. (top left)** Wake state avalanche size distributions for all mouse recordings, offset vertically for comparison of distribution shape. The points represent the original experimental data. Gray lines represent control analyses based on rate-matched Poisson surrogate data. Yellow lines show avalanche distributions from simulations of the best fit AR model. Black circles mark bins containing $\leq 5$ avalanches. The match between the yellow lines and the points shows that our AR models are good fit to the data. **(top middle)** Same as (top left), but for NREM periods. **(top right)** Same as (top left), but for REM periods. **(Bottom row)** Same as (top), but for avalanche durations. The exponent of the best fit power-law in the awake state was $1.50 \pm 0.03$ (size) and $1.56 \pm 0.07$ (duration) (mean $\pm$ s.e.m. across sessions).

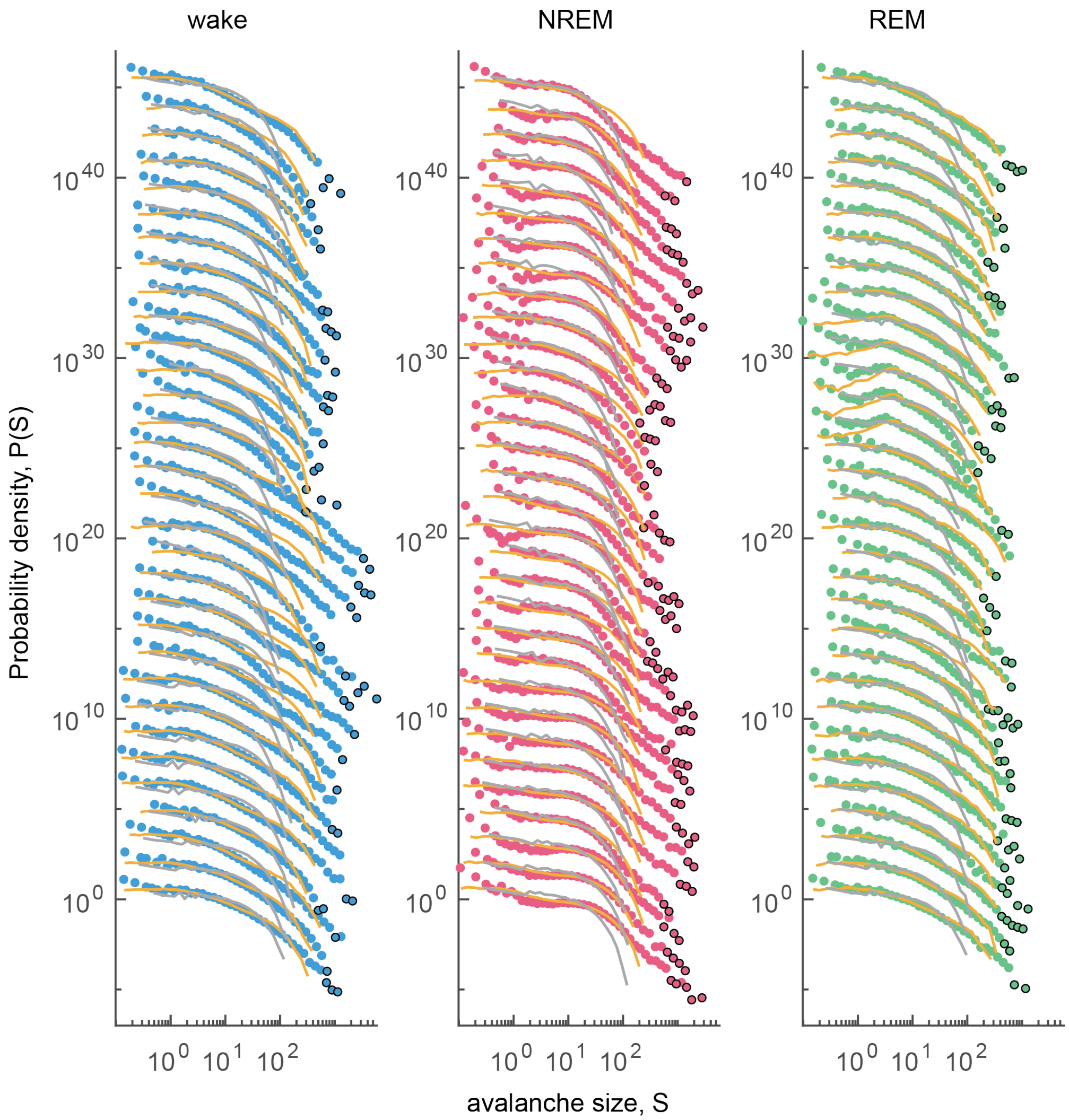


FIGURE S6. **Avalanche size distributions for rat data.** Same as top row of Fig. S5, but for rat data. The exponent of the best fit power-law in the awake state was 2.01 ± 0.07 (mean ± s.e.m. across sessions).

where $\text{diag}(S(\omega_1), \ldots, S(\omega_T))$ denotes the diagonal matrix with $S(\omega_1), \ldots, S(\omega_T)$ along the diagonal. Then defining $\tilde{\mathbf{x}} = F^\dagger \mathbf{x}$ and noticing that $F^{-1} = \frac{1}{T} F^\dagger$, we have

$$\mathbf{x}^T C^{-1} \mathbf{x} \approx \frac{1}{T} \tilde{\mathbf{x}}^\dagger \text{diag}(S(\omega_1)^{-1}, \ldots, S(\omega_T)^{-1}) \tilde{\mathbf{x}} = \frac{1}{T} \sum_{k=1}^{T} \frac{|\tilde{x}_k|^2}{S(\omega_k)} \approx \frac{1}{2\pi} \int_{-\pi}^{\pi} d\omega \, \frac{|\tilde{x}(\omega)|^2}{S(\omega)}$$

where we defined $\tilde{x}(\omega) = \sum_{t=1}^{T} x_t e^{i\omega t}$. Finally, the power spectral density of an autoregressive process is (see [11], Chapter 4)

$$S(\omega) = \frac{2\pi\sigma^2}{|1 - \tilde{\phi}(\omega)|^2}$$

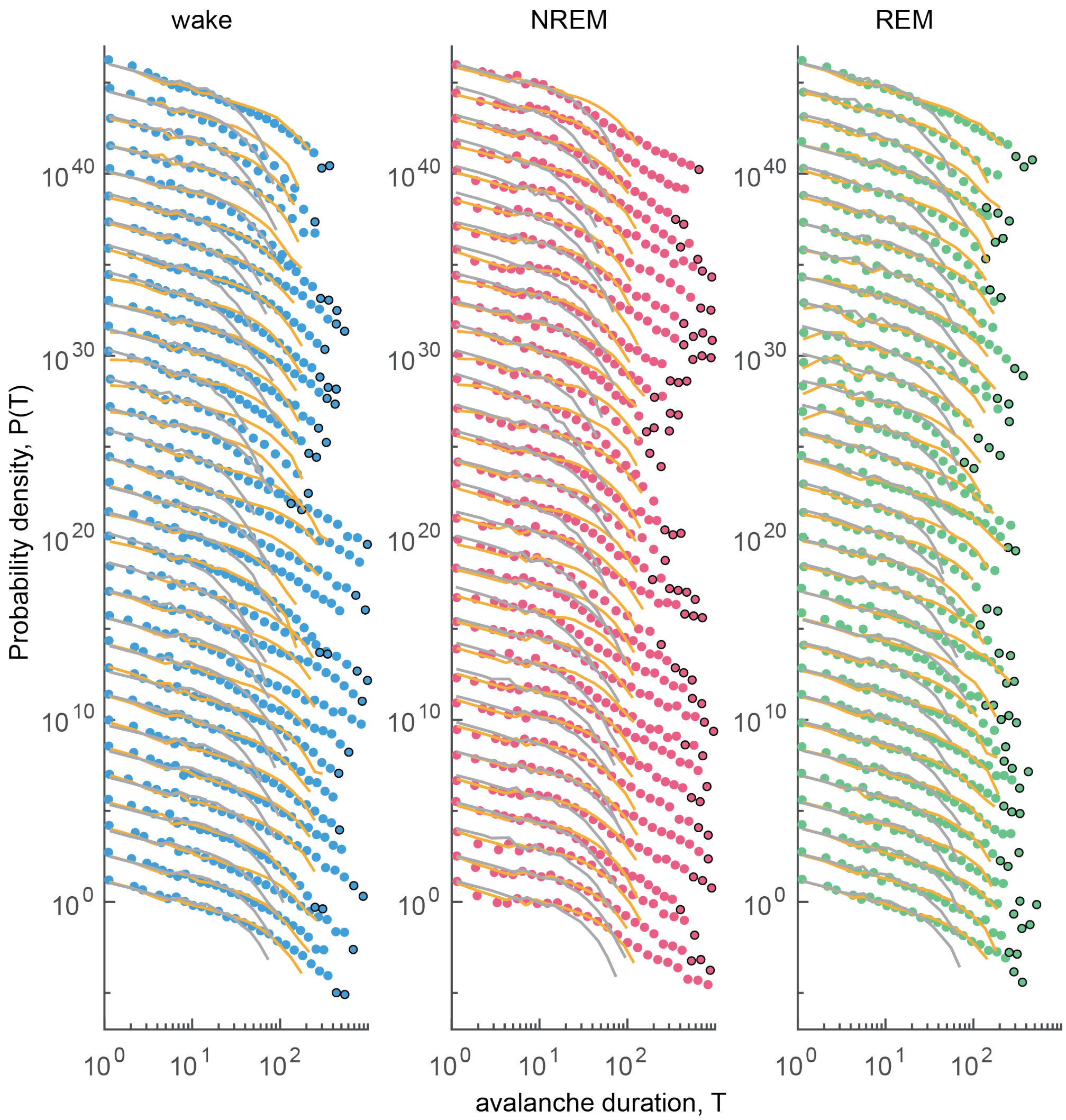


FIGURE S7. **Avalanche duration distributions for rat data.** Same as bottom row of Fig. S5, but for rat data. The exponent of the best fit power-law in the awake state was 2.32 ± 0.07 (mean ± s.e.m. across sessions).

where $\tilde{\phi}(\omega) = \sum_{t=1}^{n} \phi_n e^{-i\omega t}$, so we arrive at (Eqs. (2-3) in the main text)

$$P(\mathbf{x}) \propto \exp\left[-\frac{1}{(2\pi\sigma)^2}\int_{-\pi}^{\pi} d\omega\, |1-\tilde{\phi}(\omega)|^2 |\tilde{x}(\omega)|^2\right] \tag{3}$$

4.2. **tRG Basins of Attraction.** Fix an AR model $(\phi, \sigma)$. In Section I.C, we showed that this model flows into the $\beta$ tRG fixed point if and only if $\omega^\beta$ is the dominant term in the Taylor expansion of

$$g(\omega) = \frac{1}{(2\pi\sigma)^2}|1-\tilde{\phi}(\omega)|^2 \tag{4}$$

around $\omega = 0$. Here we derive the geometry of the sets of AR models that flow into each tRG fixed point. In other words, we locate the basins of attraction of each fixed point in the space of all AR models.

Let the Taylor expansion of $\tilde{\phi}(\omega)$ around $\omega = 0$ be $\tilde{\phi}(\omega) = \sum_{m=0}^{\infty} b_m \omega^m$. Then

$$g(\omega) \propto (1 - b_0)^2 + \mathcal{O}(\omega^2)$$

If $b_0 \neq 1$, then the dominant term in $g(\omega)$ is the constant term and the AR model flows into the trivial $\beta = 0$ fixed point. If instead $b_0 = 1$, then

$$g(\omega) \propto (-b_2 - b_2^* + b_0 b_2^* + b_2 b_0^* + |b_1|^2)\omega^2 + \mathcal{O}(\omega^4) = |b_1|^2\omega^2 + \mathcal{O}(\omega^4)$$

If $b_1 \neq 0$, then the dominant term in $g(\omega)$ is the quadratic term, so the AR model flows into the $\beta = 2$ fixed point. This is the base case of an induction argument for the following proposition:

**Proposition S4.2.1.** *For all even $\beta \geq 2$, the dominant term in $g(\omega)$ is proportional to $\omega^\beta$ if and only if $b_0 = 1$ and $b_1 = \cdots = b_{\beta/2-1} = 0 \neq b_{\beta/2}$.*

*Proof.* We already established the base case $\beta = 2$. For the induction hypothesis, suppose that the statement holds for some $\beta = k$. We want to show that the statement holds for $\beta = k + 2$. If all terms in $g(\omega)$ up to order $k - 2$ vanish, then

$$g(\omega) \propto \bigg( -b_k - b_k^* + \sum_{j=0}^{k} b_j b_{k-j}^* \bigg)\omega^k + \mathcal{O}(\omega^{k+2})$$

By the induction hypothesis, $b_0 = 1$ and $b_1 = \cdots = b_{k/2-1} = 0$, so this reduces to

$$g(\omega) \propto (-b_k - b_k^* + |b_{k/2}|^2 + b_o b_k^* + b_k b_0^*)\omega^k + \mathcal{O}(\omega^{k+2}) = |b_{k/2}|^2\omega^k + \mathcal{O}(\omega^{k+2})$$

Hence the terms up to order $k$ vanish if and only if $b_0 = 1$ and $b_1 = \cdots = b_{(k+2)/2-1} = 0$. If, in addition, $b_{(k+4)/2-1} = 0$, then the order $k+2$ term vanishes. We conclude that the dominant term in $g(\omega)$ is $\omega^{k+2}$ if and only if $b_0 = 1$ and $b_1 = \cdots = b_{(k+2)/2-1} = 0 \neq b_{(k+4)/2-1}$. That is, the statement holds for $\beta = k + 2$; by induction, it holds for all even $\beta \geq 2$. □

Next observe that

$$\tilde{\phi}(\omega) = \sum_{t=1}^{n} \phi_t e^{-i\omega t} = \sum_{m=0}^{\infty} \underbrace{\frac{(-i)^m}{m!} \sum_{t=1}^{n} \phi_t t^m}_{b_m} \omega^m$$

so we can rephrase Proposition 4.2.1 as follows: the dominant term in $g(\omega)$ is of order $\beta$ if and only if $a_0 = 1$ and $a_1 = \cdots = a_{\beta/2-1} = 0 \neq a_{\beta/2}$, where $a_m = \sum_{t=1}^{n} \phi_t t^m$. Let $A_\beta^{(n)}$ be the set of all order-$n$ history kernels that satisfy these constraints (i.e. the basin of attraction of the $\beta$ fixed point) and let

$$\hat{A}_\beta^{(n)} = \{\boldsymbol{\phi} \in \mathbb{R}^n : a_0 = 1, a_1 = \cdots = a_{\beta/2-1} = 0\}$$

Then $\hat{A}_\beta^{(n)}$ is an affine subspace of $\mathbb{R}^n$ with dimension $\max(0, n - \beta/2)$ and $A_\beta^{(n)} = \hat{A}_\beta^{(n)} \setminus \hat{A}_{\beta+2}^{(n)}$. The nested structure of the constraints translates to $\hat{A}_{\beta_2}^{(n)} \subseteq \hat{A}_{\beta_1}^{(n)}$ for all $\beta_2 \geq \beta_1$. For example, in the order-2 case, $\hat{A}_2^{(2)}$ is the line through $(0, 1)$ and $(1, 0)$, $\hat{A}_4^{(2)}$ is the singleton $\{(2, -1)\}$, and $A_2^{(2)}$ (the set order-2 AR models that flow into the $\beta = 2$ fixed point) is the line $\hat{A}_2^{(2)}$ minus the point $\hat{A}_4^{(2)}$.

A technical point is that, because the probability distribution of trajectories (Eq. (3)) that we used when performing tRG is only valid for stationary AR models, the actual tRG basins of attraction are the subsets of $\hat{A}_\beta^{(n)}$ that are on the border between stationary and nonstationary AR models; this border consists of all the history kernels $\boldsymbol{\phi}$ for which the smallest root of the characteristic polynomial $\Phi_{\boldsymbol{\phi}}(z) = \sum_{k=1}^{n} \phi_k z^k - 1$ lies on the unit circle [11]. For example, $\hat{A}_2^{(3)}$ is

a plane, but the set of AR(3) models that flows into the $\beta = 2$ fixed point is a triangular subset of this plane (main text Fig. 2F).

4.3. **KL Divergence Rate for AR models.** Fix two order-$n$ AR models A and B with parameters $(\phi_{\mathrm{A}}, \sigma_{\mathrm{A}})$ and $(\phi_{\mathrm{B}}, \sigma_{\mathrm{B}})$, respectively. We seek an analytical expression for the KL divergence rate $J(\mathrm{A} \parallel \mathrm{B})$. Let $C_T^{\mathrm{A}}$, $C_T^{\mathrm{B}}$ be the covariance matrices for a sequence of $T$ steps $\mathbf{x} = (x_1, \ldots, x_T)^T$ generated by each model. Then

$$P_{\mathrm{A}}(\mathbf{x}) = \frac{1}{(2\pi)^{T/2}|C_T^{\mathrm{A}}|^{1/2}} \exp\left(-\frac{1}{2}\mathbf{x}^T (C_T^{\mathrm{A}})^{-1}\mathbf{x}\right)$$

so that

$$\ln\left(\frac{P_{\mathrm{A}}(\mathbf{x})}{P_{\mathrm{B}}(\mathbf{x})}\right) = \frac{1}{2}\left(\ln|C_T^{\mathrm{B}}| - \ln|C_T^{\mathrm{A}}| - \mathbf{x}^T(C_T^{\mathrm{A}})^{-1}\mathbf{x} + \mathbf{x}^T(C_T^{\mathrm{B}})^{-1}\mathbf{x}\right).$$

Since $C_T^{\mathrm{A}}$ is symmetric and positive definite, its inverse is as well, and thus has a Cholesky decomposition:

$$\left(C_T^{\mathrm{A}}\right)^{-1} = R^T R$$

where $R^T$ is lower-triangular. Making the change of variables $\mathbf{y} = R\mathbf{x}$ (equivalently $\mathbf{x} = R^{-1}\mathbf{y}$), we get

$$\begin{aligned}
\int_{\mathbb{R}^T} d\mathbf{x}\, P_A(\mathbf{x}) \log\left(\frac{P_A(\mathbf{x})}{P_B(\mathbf{x})}\right) &= \frac{1}{(2\pi)^{T/2}} \int_{\mathbb{R}^T} d\mathbf{y} \exp\left(-\frac{1}{2}\mathbf{y}^T\mathbf{y}\right) \\
&\quad \times \frac{1}{2}\left(\ln|C_T^{\mathrm{B}}| - \ln|C_T^{\mathrm{A}}| - \mathbf{y}^T\mathbf{y} + \mathbf{y}^T(R^{-T}(C_T^{\mathrm{B}})^{-1}R^{-1})\mathbf{y}\right) \\
&= -\frac{1}{2}\ln\left(\frac{|C_T^{\mathrm{A}}|}{|C_T^{\mathrm{B}}|}\right) - \frac{T}{2} + \frac{1}{2(2\pi)^{T/2}} \int_{\mathbb{R}^T} d\mathbf{y} \exp\left(-\frac{1}{2}\mathbf{y}^T\mathbf{y}\right) \mathbf{y}^T(R^{-T}(C_T^{\mathrm{B}})^{-1}R^{-1})\mathbf{y} \\
&= -\frac{1}{2}\ln\left(\frac{|C_T^{\mathrm{A}}|}{|C_T^{\mathrm{B}}|}\right) - \frac{T}{2} + \frac{1}{2}\mathrm{Tr}\left((R^{-T}(C_T^{\mathrm{B}})^{-1}R^{-1})\right).
\end{aligned}$$

In the last step, we used the fact that for a real symmetric matrix $A$, if $\mathbf{y} \sim N(0, I_n)$, then $E[\mathbf{y}^T A\mathbf{y}] = \mathrm{Tr}(A)$. See [12] for a similar derivation.

Since the trace of a matrix product is invariant to cyclic permutations,

$$\mathrm{Tr}\left(R^{-T}(C_T^{\mathrm{B}})^{-1}R^{-1}\right) = \mathrm{Tr}\left((C_T^{\mathrm{B}})^{-1}R^{-1}R^{-T}\right) = \mathrm{Tr}\left((C_T^{\mathrm{B}})^{-1}(R^T R)^{-1}\right) = \mathrm{Tr}\left((C_T^{\mathrm{B}})^{-1}C_T^{\mathrm{A}}\right)$$

we have

$$\int_{\mathbb{R}^N} d\mathbf{x}\, P_{\mathrm{A}}(\mathbf{x}) \log\left(\frac{P_{\mathrm{A}}(\mathbf{x})}{P_{\mathrm{B}}(\mathbf{x})}\right) = -\frac{1}{2}\ln\left(\frac{|C_T^{\mathrm{A}}|}{|C_T^{\mathrm{B}}|}\right) - \frac{T}{2} + \frac{1}{2}\mathrm{Tr}\left((C_T^{\mathrm{B}})^{-1}C_T^{\mathrm{A}}\right). \tag{5}$$

Next, note that the covariance matrices $C_T^{\mathrm{A}}$, $C_T^{\mathrm{B}}$ are Toeplitz; that is, for any $t, t' \in \{1, \ldots, T\}$, we have $\left(C_T^{\mathrm{A}}\right)_{tt'} = C_T^{\mathrm{A}}(t - t')$ and similarly for B. Szego's theorem [13] then tells us that

$$\lim_{T\to\infty} \frac{1}{T}\sum_{k=1}^{T} H\left(\tau_{T,k}^{\mathrm{A}}\right) = \frac{1}{2\pi}\int_{-\pi}^{\pi} H\left(S^{\mathrm{A}}(\omega)\right) d\omega$$

for any function $H$ that is continuous on the range of $S^{\mathrm{A}}(\omega)$, where $S^{\mathrm{A}}(\omega)$ is the power spectral density of model A and $\tau_{T,k}^{\mathrm{A}}, k = 1, \cdots, T$ are the eigenvalues of $C_T^{\mathrm{A}}$.

Setting $H$ to the identity function, we get

$$\operatorname{Tr} C_T^{\mathrm{A}} = \sum_{k=1}^{T} \tau_{T,k}^{\mathrm{A}} \approx \frac{T}{2\pi} \int_{-\pi}^{\pi} S^A(\omega) d\omega$$

Similarly, setting $H(x) = \ln(x)$ yields

$$\ln |C_T^{\mathrm{A}}| = \ln \left( \prod_{k=1}^{T} \tau_{T,k}^{\mathrm{A}} \right) = \sum_{k=1}^{T} \ln \tau_{T,k}^{\mathrm{A}} \approx \frac{T}{2\pi} \int_{-\pi}^{\pi} \ln\, S^{\mathrm{A}}(\omega)\, d\omega$$

so that

$$\ln \left( \frac{|C_T^{\mathrm{A}}|}{|C_T^{\mathrm{B}}|} \right) \approx \frac{T}{2\pi} \left( \int_{-\pi}^{\pi} \ln\, S^{\mathrm{A}}(\omega)\, d\omega - \int_{-\pi}^{\pi} \ln\, S^{\mathrm{B}}(\omega)\, d\omega \right) = \frac{T}{2\pi} \int_{-\pi}^{\pi} \ln \left( \frac{S^{\mathrm{A}}(\omega)}{S^{\mathrm{B}}(\omega)} \right) d\omega$$

To simplify the trace term, $\operatorname{Tr}\left((C_T^{\mathrm{B}})^{-1} C_T^{\mathrm{A}}\right)$, we use the fact that the sequence of the product of Toeplitz matrices $(C_T^{\mathrm{A}}, C_T^{\mathrm{B}})$ is asymptotically Toeplitz (see [13]), and that the spectral density of the product $C_T^A C_T^B$ is (asymptotically) the product of the spectral densities $S^A$ and $S^B$; that is,

$$\lim_{T\to\infty} \frac{1}{T} \operatorname{Tr}\left(C_T^{\mathrm{A}} C_T^{\mathrm{B}}\right) = \lim_{T\to\infty} \frac{1}{T} \sum_{k=1}^{T} \rho_{T,k} = \frac{1}{2\pi} \int_{-\pi}^{\pi} S^{\mathrm{A}}(\omega) S^{\mathrm{B}}(\omega)\, d\omega$$

where $\rho_{T,k}$ are the eigenvalues of the product $C_T^{\mathrm{A}} C_T^{\mathrm{B}}$. For the inverse of such matrices,

$$\lim_{T\to\infty} \frac{1}{T} \sum_{k=1}^{T} \left(\tau_{T,k}^{\mathrm{A}}\right)^{-1} = \frac{1}{2\pi} \int_{-\pi}^{\pi} \frac{1}{S^{\mathrm{A}}(\omega)}\, d\omega.$$

Putting these two facts together gives us

$$\operatorname{Tr}\left((C_T^{\mathrm{B}})^{-1} C_T^{\mathrm{A}}\right) \approx \frac{T}{2\pi} \int_{-\pi}^{\pi} \frac{S^{\mathrm{A}}(\omega)}{S^{\mathrm{B}}(\omega)}\, d\omega$$

and thus

$$\int_{\mathbb{R}^N} d\mathbf{x}\, P_{\mathrm{A}}(\mathbf{x}) \log \left( \frac{P_{\mathrm{A}}(\mathbf{x})}{P_{\mathrm{B}}(\mathbf{x})} \right) = -\frac{1}{2} \ln \left( \frac{|C_T^{\mathrm{A}}|}{|C_T^{\mathrm{B}}|} \right) - \frac{T}{2} + \frac{1}{2} \operatorname{Tr}\left((C_T^{\mathrm{B}})^{-1} C_T^{\mathrm{A}}\right) \tag{6}$$

$$\approx -\frac{1}{2} \left( \frac{T}{2\pi} \int_{-\pi}^{\pi} \ln \left( \frac{S^{\mathrm{A}}(\omega)}{S^{\mathrm{B}}(\omega)} \right) d\omega \right) - \frac{T}{2} + \frac{1}{2} \left( \frac{T}{2\pi} \int_{-\pi}^{\pi} \frac{S^{\mathrm{A}}(\omega)}{S^{\mathrm{B}}(\omega)}\, d\omega \right) \tag{7}$$

$$= \frac{T}{4\pi} \int_{-\pi}^{\pi} \left[ \ln \left( \frac{g^{\mathrm{A}}(\omega)}{g^{\mathrm{B}}(\omega)} \right) + \frac{g^{\mathrm{B}}(\omega)}{g^{\mathrm{A}}(\omega)} - 1 \right] d\omega. \tag{8}$$

where $g^{\mathrm{A}}(\omega)$ and $g^{\mathrm{B}}(\omega)$ are defined as in Eq. (4). Rearranging the factor of $T$ and taking the $T \to \infty$ limit yields the formula for KL divergence rate in the main text.

4.4. **Numerical Minimization of KL Divergence Rate.** We have identified the tRG basins of attraction for AR models (Section 4.2) and derived a formula for the KL divergence rate between AR models (Section 4.3). To calculate $d_\beta$ for a given AR model (call it A), it remains to minimize the KL rate $J(\mathrm{A}||\mathrm{B})$ over the set of all models B in the basin of attraction of the $\beta$ fixed point. To do this, we used Matlab's nonlinear constrained optimization solver fmincon. Specifically, we parameterized $\hat{A}_\beta^{(n)}$ by the first $n-\beta/2$ coefficients of the history kernel (these are enough to uniquely identify a point on $\hat{A}_\beta^{(n)}$ since it has dimensionality $n-\beta/2$). We initialized the optimization process at the point on $\hat{A}_\beta^{(n)}$ that was closest in Euclidean distance to the history kernel of model A. To

ensure that the optimizer stayed on the critical manifold (which is a subset of $\hat{A}_\beta^{(n)}$; see Section 4.2), we used the inequality constraint

$$|\text{smallest root of char. poly. of B}| \geq 1 - \epsilon$$

where $\epsilon = 10^{-2}$ is a numerical tolerance. For complete implementation details, see our publicly available toolkit at `https://github.com/sam-sooter/prox_crit_toolkit.`.

4.5. **Further details on AR model estimation and $d_2$ error bars.** In the time-resolved $d_2$ analysis in Fig. S1 and main text Fig. 4H, we fitted AR models to time series by maximum likelihood estimation. Here we detail this procedure in the general case of a segmented time series $\{x_t^{(i)} : 1 \leq i \leq W, 1 \leq t \leq T^{(i)}\}$ (here $x_t^{(i)}$ is the value of the time series at time step $t$ during segment $i$, $W$ is the number of segments, and $T^{(i)}$ is the duration of the $i$th segment), although in Fig. 4H the time series were not segmented (i.e. $W = 1$). For a given AR model order $n$, we maximized the log-likelihood

$$\mathcal{L}(\{x_t^{(i)}\}|\boldsymbol{\phi}, \sigma) = -\sum_{i=1}^{W} \left[ \frac{T^{(i)}}{2} \log(2\pi\sigma^2) + \frac{1}{2\sigma^2} \sum_{t=1}^{T^{(i)}} \left( x_t^{(i)} - \sum_{l=1}^{\min(n,t-1)} \phi_l x_{t-l}^{(i)} \right)^2 \right]$$

First, fix $\boldsymbol{\phi}$ and find the value of $\sigma$ (call it $\sigma^*$) that maximizes the likelihood:

$$\frac{\partial \mathcal{L}}{\partial \sigma}\bigg|_{(\boldsymbol{\phi}, \sigma^*)} = \sum_{i=1}^{W} \left[ -\frac{T^{(i)}}{\sigma^*} + \frac{1}{\sigma^{*3}} \sum_{t=1}^{T^{(i)}} \left( x_t^{(i)} - \sum_{l=1}^{\min(n,t-1)} \phi_l x_{t-l}^{(i)} \right)^2 \right] = 0$$

$$\implies \quad \sigma^* = \sqrt{\frac{1}{T} \sum_{i=1}^{W} \sum_{t=1}^{T^{(i)}} \left( x_t^{(i)} - \sum_{l=1}^{\min(n,t-1)} \phi_l x_{t-l}^{(i)} \right)^2}$$

Here we introduced $T := \sum_{i=1}^{W} T^{(i)}$. Substituting this back into the likelihood function yields

$$\mathcal{L}(\{x_t^{(i)}\}|\boldsymbol{\phi}, \sigma^*) = -\frac{T}{2} \left[ \log \left( \frac{2\pi}{T} \sum_{i=1}^{W} \sum_{t=1}^{T^{(i)}} \left( x_t^{(i)} - \sum_{l=1}^{\min(n,t-1)} \phi_l x_{t-l}^{(i)} \right)^2 \right) + 1 \right]$$

Maximizing this likelihood function is equivalent to minimizing the sum of squared errors. We do this in the usual way; specifically, for each $i = 1, \ldots, W$, let $X^{(i)}$ be the $T^{(i)} \times n$ matrix with entries

$$X_{tk}^{(i)} = \begin{cases} x_{t-k}^{(i)} & t - k > 0 \\ 0 & t - k \leq 0 \end{cases}$$

and let $\mathbf{y}^{(i)} = (x_1^{(i)}, \ldots, x_{T^i}^{(i)})^T$. Then the maximum likelihood history kernel is

$$\boldsymbol{\phi}^* = (X^T X)^{-1} X^T \mathbf{y}$$

where

$$X = \begin{pmatrix} X^{(1)} \\ \vdots \\ X^{(W)} \end{pmatrix}; \quad \mathbf{y} = \begin{pmatrix} \mathbf{y}^{(1)} \\ \vdots \\ \mathbf{y}^{(W)} \end{pmatrix}$$

Next, to obtain uncertainties for the estimated history kernel, we compute the Hessian of the log-likelihood,

$$H_{jk}(\phi^*) := \frac{\partial^2 \mathcal{L}(\{x_t^{(i)}\}|\boldsymbol{\phi}, \sigma^\star)}{\partial \phi_j \partial \phi_k}\Big|_{\boldsymbol{\phi}=\boldsymbol{\phi}^*} = T\left[\frac{2B_j(\boldsymbol{\phi}^*)B_k(\boldsymbol{\phi}^*)}{A(\boldsymbol{\phi}^*)^2} - \frac{1}{A(\boldsymbol{\phi}^*)}\sum_{i=1}^{W}\sum_{t=\max(j,k)+1}^{T^{(i)}} x_{t-j}^{(i)} x_{t-k}^{(i)}\right]$$

where

$$A(\boldsymbol{\phi}^*) = \sum_{i=1}^{W}\sum_{t=1}^{T^{(i)}}\left(x_t^{(i)} - \sum_{j=1}^{\min(n,t-1)} \phi_l^* x_{t-l}^{(i)}\right)^2; \quad B_j(\boldsymbol{\phi}^*) = \sum_{i=1}^{W}\sum_{t=j+1}^{T^{(i)}}\left(x_t^{(i)} - \sum_{l=1}^{\min(n,t-1)} \phi_l^* x_{t-l}^{(i)}\right) x_{t-j}^{(i)}$$

The matrix $H(\boldsymbol{\phi}^*)$ encodes the quadratic approximation of the log-likelihood function around its minimum at $\boldsymbol{\phi}^*$. Moreover, if we treat the likelihood (of the data, given a particular history kernel) as the likelihood of the kernel given the data (this is equivalent to adopting a uniform prior on the kernel), then the standard deviation of $d_2(\boldsymbol{\phi})$ is

$$\begin{aligned}\Delta d_2(\boldsymbol{\phi}) = \sqrt{\langle (d_2(\boldsymbol{\phi}) - \langle d_2(\boldsymbol{\phi})\rangle)^2\rangle} &\approx \sqrt{\langle (d_2(\boldsymbol{\phi}^*) + (\nabla d_2(\boldsymbol{\phi}^*))^T(\boldsymbol{\phi} - \boldsymbol{\phi}^*) - d_2(\boldsymbol{\phi}^*))^2\rangle} \\ &= \sqrt{(\nabla d_2(\boldsymbol{\phi}^*))^T \langle (\boldsymbol{\phi} - \boldsymbol{\phi}^*)(\boldsymbol{\phi} - \boldsymbol{\phi}^*)^T\rangle \nabla d_2(\boldsymbol{\phi}^*)} \\ &\approx \sqrt{-(\nabla d_2(\boldsymbol{\phi}^*))^T H^{-1} \nabla d_2(\boldsymbol{\phi}^*)} \qquad (9)\end{aligned}$$

where $\langle\cdot\rangle$ denotes an average over the likelihood of the kernel given the data. We used Eq. (9) to compute the $d_2$ error bars shown in Fig. 4.

## 5. Model Benchmarking

In the main text Section II and Fig. 3, we showed that $d_2$ faithfully represented the ground truth distance to criticality for three test case models. Here we detail this process more fully.

5.1. **Numerical calculation of KL divergence rate.** Much of this section relies on numerically calculating KL divergence rates between pairs of models, so we will first describe this procedure in general terms. Consider a family of time series models, with each model corresponding to a list of parameters $\theta$ and a conditional distribution $P_\theta(x_t|x_{t-1}, x_{t-2}, \dots) \equiv P_\theta(x_t|\text{past})$. We want to calculate the KL divergence rate $J(\theta_\text{A}||\theta_\text{B})$ between two models in this family with parameter values $\theta_\text{A}$ and $\theta_\text{B}$. In lieu of an analytical expression, we use the following numerical procedure:

(1) Run model A for $T$ time steps by repeatedly sampling the distribution $P_{\theta_\text{A}}(x_t|\text{past})$.
(2) Repeat this $N$ times to generate an ensemble of time series $x_1^{(i)}, x_2^{(i)}, \dots, x_T^{(i)}$ $(i = 1, 2, \dots, N)$, each of length $T$.
(3) Calculate the log-likelihoods

$$\mathcal{L}_A^{(i)} = \log\left[\prod_{t=1}^{T} P_A(x_t^{(i)}|\text{past})\right]; \mathcal{L}_B^{(i)} = \log\left[\prod_{t=1}^{T} P_B(x_t^{(i)}|\text{past})\right]$$

for each time series under models A and B.
(4) Estimate

$$J(\theta_A||\theta_B) \approx \frac{1}{TN}\sum_{i=1}^{N}\left(\mathcal{L}_A^{(i)} - \mathcal{L}_B^{(i)}\right) \qquad (10)$$

In the limit of large $T$ and large $N$, this estimate becomes exact.

5.2. **Univariate Hawkes Process.** Since real neural activity is discretized into point events ("spikes"), we considered a univariate Hawkes process. In this model, spikes are modeled as a Poisson process with time-varying rate

$$\lambda(t) = \mu + A \sum_{t_i < t} \phi(t - t_i)$$

where the sum is over all spike times earlier than $t$, $\mu$ is a background spiking rate, and $\phi(t)$ is a history kernel (Fig. 3A). For simplicity, we choose $\phi(t) = e^{-t}$. Regardless of the value of $\mu$, the model is stable for $A < 1$ and unstable for $A > 1$; at the transition point $A = A_c = 1$, the spiking statistics are scale-invariant [3], thus the model is at criticality.

Our definition of distance to criticality for time series models was applied here with some changes. First, imagine running a Hawkes process with parameters $A, \mu$ until it generates $K$ spikes. Let the log-likelihood that these spikes occur at times $t_1, \ldots, t_K$ be $\mathcal{L}_{A,\mu}(t_1, \ldots, t_K)$. (We derive a simple expression for this likelihood function in Prop. 5.2.1.) Then the KL divergence per unit time from the model $A, \mu$ to a critical model $A_c, \mu_c$ is

$$J(A, \mu || A_c, \mu_c) := \lim_{K \to \infty} \left\langle \frac{1}{t_K} \frac{\mathcal{L}_{A,\mu}(t_1, \ldots, t_K)}{\mathcal{L}_{A_c,\mu_c}(t_1, \ldots, t_K)} \right\rangle_{A,\mu} \tag{11}$$

where $\langle \cdot \rangle_{A,\mu}$ denotes an average over realizations of the model with parameters $A, \mu$. As before (cf main text Eq. (1)), distance to criticality is the minimum of this KL divergence rate taken over the set of critical models. In this case, this means minimizing over $\mu_c$. We calculated the expression in Eq. (11) with the numerical procedure described in Section 5.1. Specifically, we draw many sequences $t_1, \ldots, t_K$ of spike times from the model with parameters $A, \mu$ using the algorithm of [14], calculate the quantity inside the angled brackets in Eq. (11) for each draw, and average over draws. We then perform a grid search for the optimal $\mu_c$ (Fig. 3C). The results of this calculation are the black points shown as a function of $A$ in Fig. 3B.

Can an AR model-based approach recover the ground-truth distance to criticality? For a given realization of a Hawkes process, we counted the number of spikes in time bins of length $\Delta t$ and then fitted an AR model to the resulting spike-count time series. The best-fit AR model's distance to criticality closely agrees with the ground-truth value obtained from Eq. (11) across a wide range of values of $A$ (Fig. 3B,C).

**Proposition S5.2.1.** *For the Hawkes process with parameters* $(A, \mu)$*, the probability distribution for the next spike time given the spiking history* $t_{i-1}, t_{i-2}, \ldots$ *is*

$$P(t_i | t_{i-1}, t_{i-2}, \ldots) = exp\left[(\lambda_{i-1} - \mu)(e^{-(t_i - t_{i-1})} - 1) - \mu(t_i - t_{i-1})\right] \left((\lambda_{i-1} - \mu) e^{-(t_i - t_{i-1})} + \mu\right)$$

*where* $\lambda_{i-1}$ *is the rate immediately after the spike at time* $t_{i-1}$*. The log-likelihood of a sequence of spike times (given an initial spike at* $t_0 = 0$*) is then*

$$\mathcal{L}(t_1, \ldots, t_N) = \sum_{i=1}^{N} \ln P(t_i - t_{i-1} | t_{i-1}, t_{i-2}, \ldots, t_0)$$

*Proof.* First, calculate the likelihood that the next spike occurs later than time $\tau$ after the most recent spike at time $t_{i-1}$:

$$P(t_i - t_{i-1} \geq \tau | t_{i-1}, t_{i-2}, \dots) = \lim_{\Delta \to 0} \prod_{k=1}^{\lceil \tau/\Delta \rceil} (1 - \lambda(t_{i-1} + k\Delta)\Delta) = \lim_{\Delta \to 0} \prod_{k=1}^{\lceil \tau/\Delta \rceil} \exp\left(-\lambda(t_{i-1} + k\Delta)\Delta\right)$$
$$= \lim_{\Delta \to 0} \exp\left(-\sum_{k=1}^{\lceil \tau/\Delta \rceil} \lambda(t_{i-1} + k\Delta)\Delta\right) = \exp\left(-\int_{t_{i-1}}^{t_{i-1}+\tau} \lambda(t)\, dt\right)$$

Here $\lambda(t)$ is the rate at time $t$ given that there were no spikes between $t_{i-1}$ and $t$. It is easy to show (taking advantage of the property $\frac{d\phi(t)}{dt} = -\phi(t)$ of the exponential kernel) that

$$\lambda(t) = (\lambda_{i-1} - \mu)e^{-(t - t_{i-1})} + \mu \tag{12}$$

so

$$P(t_i - t_{i-1} \geq \tau | t_{i-1}, t_{i-2}, \dots) = \exp\left[(\lambda_{i-1} - \mu)(e^{-\tau} - 1) - \mu\tau\right]$$

Differentiating with respect to $\tau$ then yields the desired expression. □

5.3. **Nonlinear model: Langevin dynamics in quartic potential.** Next, we tested our distance to criticality pipeline on a nonlinear time series model, the overdamped Langevin equation in a quartic potential:

$$\dot{x} = -bx^2 - cx^3 + \xi(t) \tag{13}$$

where as usual $\xi$ is Gaussian white noise with $\langle \xi(t) \rangle = 0$ and $\langle \xi(t)\xi(t') \rangle = \sigma^2 \delta(t - t')$. For what values of the parameters $(b, c, \sigma)$ is this model at criticality? Since there is a transition from stable to unstable dynamics at $c = 0$ regardless of the values of $b$ and $\sigma$, an initial guess might be that the half-plane of models $\{(b, c, \sigma) : c = 0, \sigma > 0\}$ are all at criticality. However, if $b \neq 0$, then scale-invariance does *not* emerge as $c \to 0^+$. Instead, the model exhibits weak, short timescale fluctuations around the fixed point $x^* = b/c$ of the deterministic dynamics. This can be seen by changing variables to $z = x - x^*$:

$$\dot{z} = -\frac{b^2}{c} z + 2bz^2 - cz^3 + \xi$$

If $b \neq 0$, then as $c \to 0^+$, the coefficient of the linear term approaches $-\infty$, making the timescale of fluctuations in $z$ approach zero. Hence the only critical models in this family are the continuous-time random walks $\mathcal{M}_{\text{crit}} = \{(b, c, \sigma) : b = c = 0\}$.

To calculate the ground-truth distance to criticality for models in this family, we followed the same numerical strategy as for the Hawkes process, except with a time series instead of spikes. Specifically, we simulate Eq. (13) in discrete time steps of size $\Delta t$ and calculate the average (over many simulations) of the log-likelihood ratio between the true model $(b, c, \sigma)$ and critical models $(b_c = 0, c_c = 0, \sigma_c)$, per unit time; we then choose the value of $\sigma_c$ that minimizes this quantity. Repeating this procedure for various values of $\Delta t$ shows that the measured distance to criticality limits to a finite value as $\Delta t \to 0$. Moreover, applying the AR model pipeline to sample time series produced by the model Eq. (13) closely reproduces the ground-truth distance to criticality (Fig. 3E). This example illustrates the importance of a non-parametric definition of distance to criticality in systems with more than one relevant parameter, as it is not at all clear a priori how to combine $b$ and $c$ to measure deviation from the critical point at $b = c = 0$.

To calculate the KL divergence rate between a given model $(b, c, \sigma)$ and the models in $\mathcal{M}_{\text{crit}}$, we approximate Eq. (13) with the discrete update equation

$$x(t+\Delta t) = x(t) - (bx(t)^2 + cx(t)^3)\Delta t + y(t)\sigma\sqrt{\Delta t} \tag{14}$$

where $y(t) \sim \mathcal{N}(0,1)$ and is drawn independently for each $t$ [cite]. The probability distribution for $x(t+\Delta t)$ given $x(t)$ under the discrete dynamics Eq. (14) is

$$P(x(t+\Delta t)|x(t)) = \frac{1}{\sqrt{2\pi\sigma^2\Delta t}} \exp\left\{-\frac{1}{2\sigma^2\Delta t}\left[x(t+\Delta t) - (x(t) - \Delta t(bx(t)^2 + cx(t)^3))\right]^2\right\} \tag{15}$$

so the log-likelihood of a $T$-step discrete trajectory $x(t_0+\Delta t), x(t_0+2\Delta t), \ldots, x(t_0+T\Delta t)$ given the starting point $x(t_0)$ is

$$\mathcal{L}(x_1, \ldots, x_T|x_0) = \sum_{k=1}^{T} \log P(x_k|x_{k-1}) \tag{16}$$

where for compactness we abbreviate $x_k := x(t_0 + k\Delta t)$. To calculate the KL divergence rate from the model $A = (b, c, \sigma)$ to a critical model $B = (0, 0, \sigma_c) \in \mathcal{M}_{\text{crit}}$, we follow the procedure described in Section 3. First, we draw $N$ trajectories $x_1^{(i)}, \ldots, x_T^{(i)}$ $(i = 1, \ldots, N)$ of duration $T$ steps from model $A$ with Eq. (14). Second, we use Eq. (16) to calculate the log-likelihoods $\mathcal{L}_A^1, \ldots, \mathcal{L}_A^N$ and $\mathcal{L}_B^1, \ldots, \mathcal{L}_B^N$ of these trajectories under $A$ and $B$, respectively. Then Eq. (10) becomes

$$J(A||B) \approx \frac{1}{NT\Delta t}\sum_{i=1}^{N}\sum_{k=1}^{T}\Bigg\{\ln\left(\frac{\sigma_c}{\sigma}\right) - \frac{1}{2\sigma^2\Delta t}\left[x_k^{(i)} - \left(x_{k-1}^{(i)} - \Delta t\left(b{x_{k-1}^{(i)}}^2 + c{x_{k-1}^{(i)}}^3\right)\right)\right]^2 \tag{17}$$

$$+ \frac{1}{2\sigma_c^2\Delta t}\left[x_k^{(i)} - x_{k-1}^{(i)}\right]^2\Bigg\} \tag{18}$$

where we added the extra factor of $1/\Delta t$ to put $J$ in units of nats per time (rather than nats per time step). To calculate distance to criticality, we minimize $J(A||B)$ over $\sigma_c$:

$$\frac{\partial J(A||B)}{\partial \sigma_c} = 0 \implies \frac{1}{\sigma_c\Delta t} - \frac{1}{NT(\Delta t)^2\sigma_c^3}\sum_{i=1}^{N}\sum_{k=1}^{T}\left[x_k^{(i)} - x_{k-1}^{(i)}\right]^2 = 0$$

$$\implies \sigma_c = \sqrt{\frac{1}{NT\Delta t}\sum_{i=1}^{N}\sum_{k=1}^{T}\left[x_k^{(i)} - x_{k-1}^{(i)}\right]^2}$$

Plugging this expression into Eq. (18) then yields the distance to criticality.

5.4. **Bursty model: Langevin dynamics with multiplicative noise.** In addition to spiking and nonlinearity, another feature of neural systems that is absent in AR models is burstiness. In awake, adult animals, brain activity is not bursty, as we will show in the next section. However, in other situations, brain dynamics can be rather bursty. Examples include in vitro systems [15, 16, 17, 18], some anesthetized states [19], and newborn animals [20, 21]. AR models, on the other hand, produce time series that are symmetric about their mean and have no definite floor. As a third test case, we considered a simple model with multiplicative noise that captures both bursty and non-bursty dynamics,

$$\dot{x} = -bx + h + \sqrt{x}\xi \tag{19}$$

where as before $\xi$ is Gaussian white noise with $\langle\xi(t)\rangle = 0$ and $\langle\xi(t)\xi(t')\rangle = \sigma^2\delta(t-t')$. When $h$ (which can be interpreted as external drive) is close to zero, this model exhibits silence-separated bursts because of the $\sqrt{x}$ multiplicative noise. Increasing $h$ moves the stable fixed point at $x = h/b$

to larger $x$, making the dynamics less bursty. For any $h \geq 0$, the model transitions from stable to unstable dynamics and exhibits scale-invariance at $b = 0$. Hence the critical models in this family are those on the half-line $b = 0, h \geq 0$.

We calculated ground-truth distance to criticality in the same way as for the model Eq. (13), except that here there are two parameters ($h_c$ and $\sigma_c$) to vary when minimizing the KL divergence rate over the set of critical models (Fig. 3F). In brief, we simulated Eq. (19) in discrete time steps by sampling the exact solution to the associated Fokker-Planck equation, as described in [22]. (A naive Euler-type update equation similar to Eq. (14) is inadequate because of the $\sqrt{x}$ multiplicative noise.) We then again used the solution to the Fokker-Planck equation given in [22] to compute the likelihood of the simulated trajectories under the actual model (parameters $(b, h, \sigma)$) and a critical model (parameters $(b_c = 0, h_c, \sigma_c)$). Next, we plugged the result into (10) to compute the KL divergence rate from the model $(b, h, \sigma)$ to the critical model $(b_c = 0, h_c, \sigma_c)$. Finally, we performed a grid search to find the $h_c$ and $\sigma_c$ that minimized the KL rate.

Comparing the ground-truth distance to criticality to that obtained via AR model fits reveals close agreement across a large swath of values of $b$ and $h$ (Fig. 3G). This agreement breaks down as $h \to 0^+$ and the dynamics become more bursty. Put simply, our approach is robust to low levels of burstiness, but breaks down for very bursty data.

## 6. Dependence on Time Bin Length and AR Model Order

When working with spike data, how should one choose the time bin $\Delta t$ for counting spikes and the model order for calculating $d_2$? These are the two data- analytic parameter choices that most impact the values of $d_2$. One important fact that helps clarify this choice is demonstrated in Supplementary Fig. S8. We show that two $d_2$ measurements are comparable if model order and $\Delta t$ are chosen such that their product is fixed. The product (model order) $\times \Delta t$ is the "temporal reach" (in seconds) of the AR model history kernel. If this temporal reach is the same for two different $d_2$ measurements, they can be fairly compared. Otherwise, comparisons should not be made.

## 7. Relating $d_2$ to the longest time scale of the dynamics

Many previously established data analytic methods assess the dominant time scale of a time series. These include estimates of the autocorrelation time [23, 24, 25] and other methods. Such methods are expected to be related to proximity to criticality because dominant timescales diverge as a system approaches criticality - a phenomenon sometimes called 'critical slowing down'. Thus, we expect $d_2$ to be related to the dominant time scale. However, we show in Supplementary Fig. S9 that true proximity to criticality (i.e. $d_2$) is not simply a readout of the longest time scale of a system. We randomly generated many AR(5) models all with the same maximum time scale. Here the maximum time scale is set by the root of the characteristic polynomial of the AR model that is closest to the unity circle. We found that the variability in $d_2$ for fixed maximum time scale was comparable to the difference in average $d_2$ caused by increasing the maximum time scale by 20 time steps. Thus, we conclude that the longest time scale does not determine proximity to criticality, at least in AR models and suggests that $d_2$ will better capture proximity to criticality than methods that rely solely on measuring the dominant time scale.

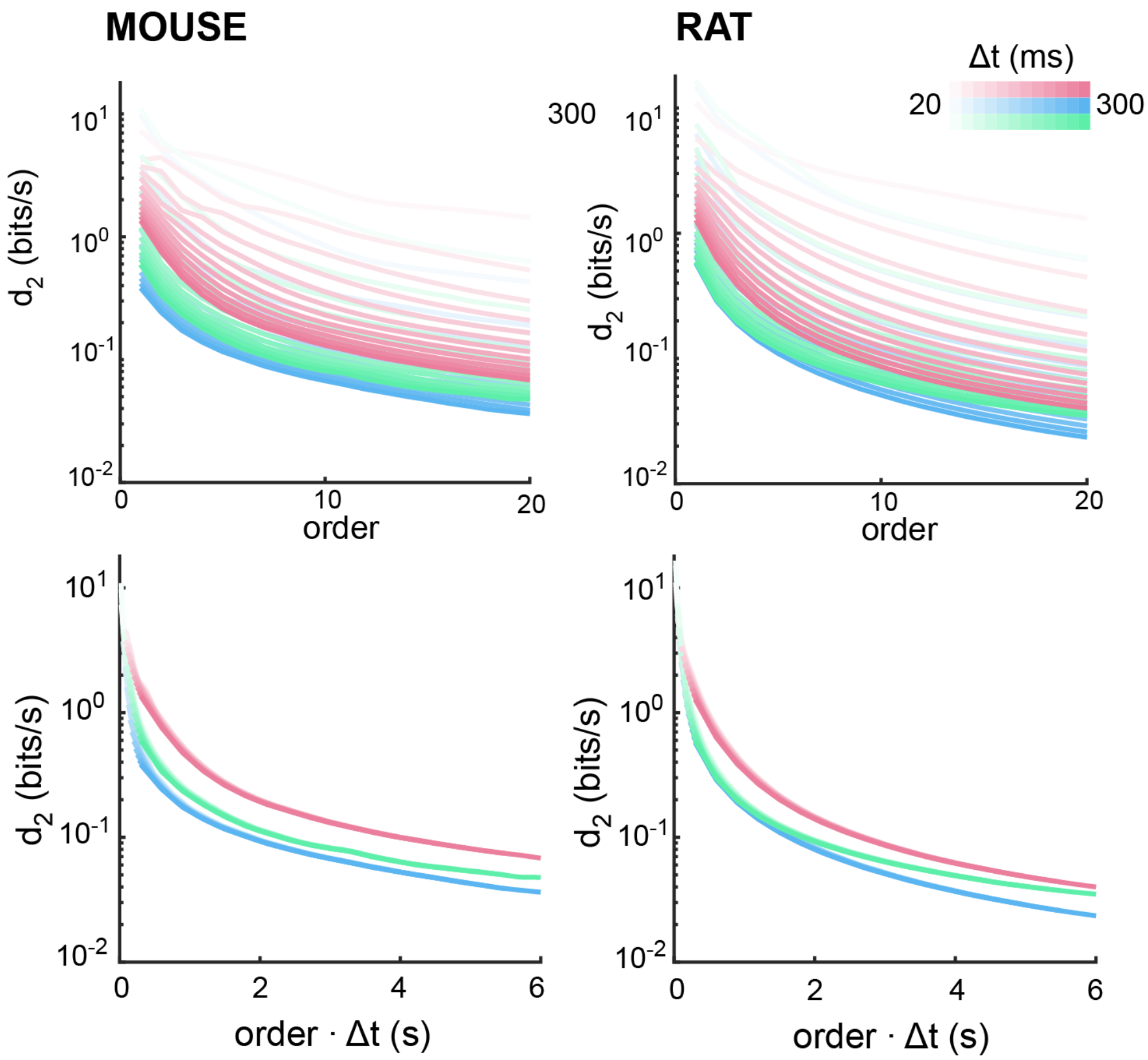


FIGURE S8. **Temporal reach explains dependence of $d_2$ on time bin and model order.** In general, when applying our approach to spike data, $d_2$ will depend strongly on the choice of time bin $\Delta t$ used to count spikes and the choice of AR model order. The top row shows how $d_2$ varies with model order and $\Delta t$ for the mouse data (left) and rat data (right) and each sleep/wake state (blue - wake, red - NREM, green - REM). The wide ranging $d_2$ values collapse onto a single function when considered in terms of "temporal reach", i.e. the product of (model order) $\times \Delta t$. The temporal reach is the time (in seconds) that the AR model kernel reaches into the past.

## 8. Restricting the set of critical models to its intersection with a tractable family

Directly implementing our definition of distance to criticality (main text Eq. (1)) would require knowing the structure of the abstract set $\mathcal{M}_c$ of *all* critical models. As a practical compromise, we restricted our attention (main text Section I.C) to the critical models lying within a tractable family of models, AR models. In doing so, we assumed that the nearest critical model (in the sense of KL rate; call this model B*) to the model whose DTC we are computing (call it A) can be approximated by a critical AR model; that is, $B^* \approx B$ for some critical AR model B. More

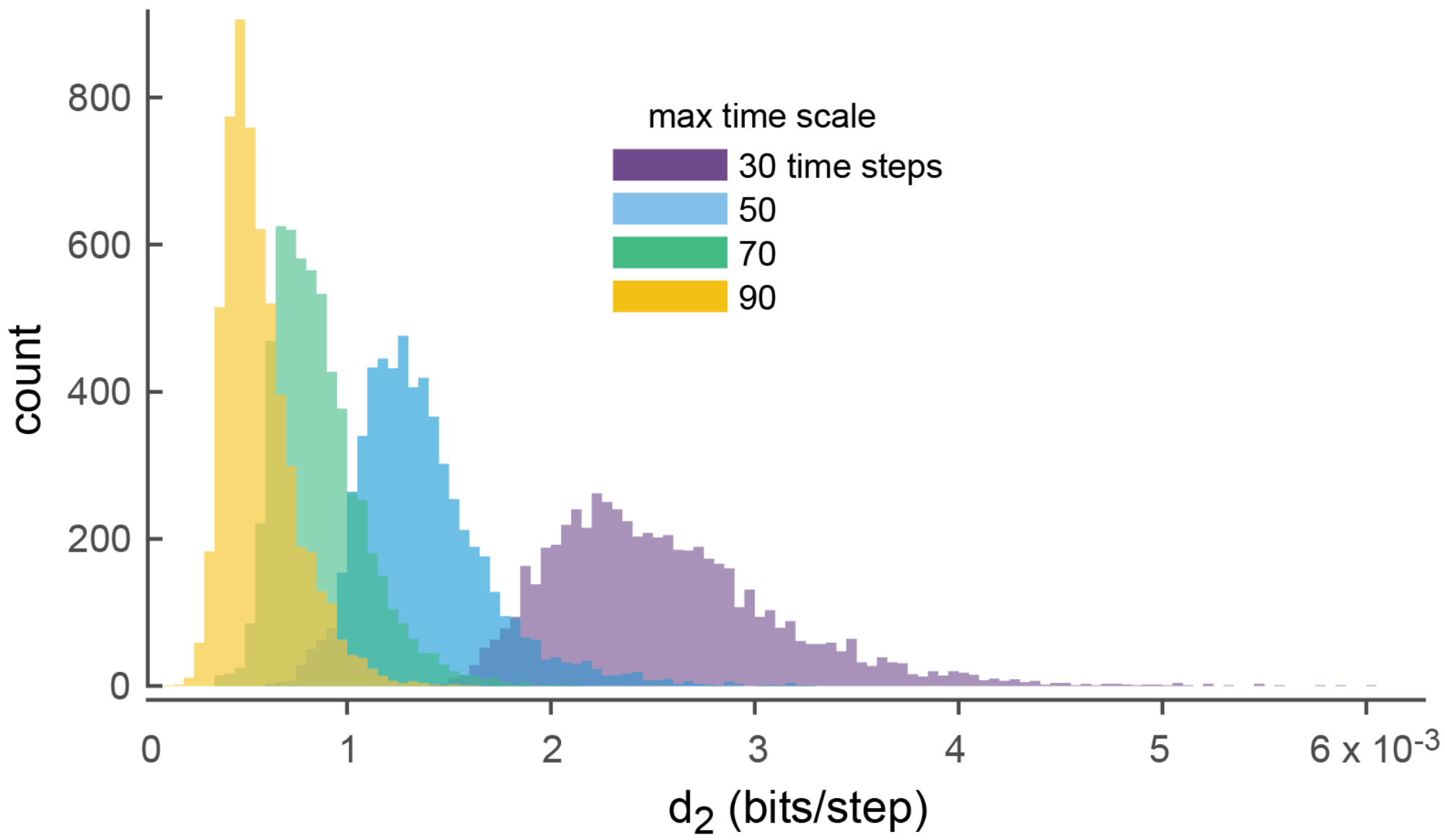


FIGURE S9. **Distance to criticality ($d_2$) is not solely determined by the longest time scales of a system.** We considered four ensembles of AR(5) models, each with a fixed maximum time scale (color indicates max time scale). For fixed max time scale, distance to criticality $d_2$ can vary by as much as an order of magnitude. Here, $d_2$ was calculated analytically from the AR history kernel. Thus, the variability in $d_2$ for fixed time scale is not due to statistical noise in simulations.

work is needed to understand when this approximation is valid; intuitively, however, if A is well-approximated by an AR model, then B* should be well-approximated by a critical AR model. We applied the same logic to the familes of models used for benchmarking (main text Section II; Section S3); when calculating the "ground truth" distance to criticality of a model in one of these families (e.g. Hawkes processes), we limited the KL rate minimization to critical models lying in the same family. This is admittedly pragmatic and not formally justified. A more flexible implementation of our definition of distance to criticality that does not rely on any specific class of models will be a target of future work.

## References


[1] Sabrina A Jones, Jacob H Barfield, V Kindler Norman, and Woodrow L Shew. Scale-free behavioral dynamics directly linked with scale-free cortical dynamics. 12:e79950. ISSN 2050-084X. doi: 10.7554/eLife.79950. URL `https://doi.org/10.7554/eLife.79950`. Publisher: eLife Sciences Publications, Ltd.

[2] Keith B. Hengen and Woodrow L. Shew. Is criticality a unified setpoint of brain function? *Neuron*, 113:1–17, 2025. ISSN 0896-6273. doi: 10.1016/j.neuron.2025.05.020.

[3] Antonio J. Fontenele, J. Samuel Sooter, V. Kindler Norman, Shree Hari Gautam, and Woodrow L. Shew. Low-dimensional criticality embedded in high-dimensional awake brain dynamics. *Science Advances*, 10(17):eadj9303, 2024. doi: 10.1126/sciadv.adj9303. URL `https://www.science.org/doi/10.1126/sciadv.adj9303`.

[4] Nir Friedman, Shinya Ito, Braden A. W. Brinkman, Masanori Shimono, R. E. Lee DeVille, Karin A. Dahmen, John M. Beggs, and Thomas C. Butler. Universal critical dynamics in high resolution neuronal avalanche data. *Physical Review Letters*, 108(20):208102, 2012. ISSN 0031-9007, 1079-7114. doi: 10.1103/PhysRevLett.108.208102. URL `https://link.aps.org/doi/10.1103/PhysRevLett.108.208102`.

[5] Woodrow L. Shew, Wesley P. Clawson, Jeff Pobst, Yahyah Karimipanah, Nathaniel C. Wright, and Ralf Wessel. Adaptation to sensory input tunes visual cortex to criticality. *Nature Physics*, 11(8):659–663, jun 2015. ISSN 1745-2473. doi: 10.1038/nphys3370. URL `http://www.nature.com/doifinder/10.1038/nphys3370`.

[6] Zhengyu Ma, Gina G. Turrigiano, Ralf Wessel, and Keith B. Hengen. Cortical circuit dynamics are homeostatically tuned to criticality in vivo. *Neuron*, 104(4):655–664.e4, 2019. ISSN 1097-4199. doi: 10.1016/j.neuron.2019.08.031.

[7] James N. McGregor, Clayton A. Farris, Sahara Ensley, Aidan Schneider, Leandro J. Fosque, Chao Wang, Elizabeth I. Tilden, Yuqi Liu, Jianhong Tu, Halla Elmore, Keenan D. Ronayne, Ralf Wessel, Eva L. Dyer, Kiran Bhaskaran-Nair, David M. Holtzman, and Keith B. Hengen. Failure in a population: Tauopathy disrupts homeostatic set-points in emergent dynamics despite stability in the constituent neurons. *Neuron*, 112(21):3567–3584.e5, 2024. ISSN 0896-6273. doi: 10.1016/j.neuron.2024.08.006. URL `https://www.cell.com/neuron/abstract/S0896-6273(24)00578-6`.

[8] Antonio J Fontenele, J Samuel Sooter, Ehsan Ziarati, Andrea K Barreiro, Cheng Ly, and Woodrow Shew. Is critical brain dynamics more prevalent than previously thought? 2025. doi: 10.1101/2025.09.02.673722.

[9] Daniel B. Larremore, Woodrow L. Shew, Edward Ott, Francesco Sorrentino, and Juan G. Restrepo. Inhibition causes ceaseless dynamics in networks of excitable nodes. *Physical Review Letters*, 112(13):138103, 2014. ISSN 0031-9007. doi: 10.1103/physrevlett.112.138103.

[10] Jingwen Li and Woodrow L. Shew. Tuning network dynamics from criticality to an asynchronous state. *PLoS Computational Biology*, 16(9):e1008268, 2020. ISSN 1553-734X. doi: 10.1371/journal.pcbi.1008268.

[11] Robert H. Shumway and David S. Stoffer. *Time Series Analysis and its Applications*. Springer, 4th edition, 2006.

[12] Eric Grivel, Roberto Diversi, and Fernando Marchan. Kullback-Leibler divergence and Renyi divergence rate for Gaussian stationary ARMA processes comparison. *Digital Signal Processing*, 116:103089, 2021.

[13] Robert M. Gray. Toeplitz and circulant matrices: A review. *Foundations and Trends in Communications and Information Theory*, 2(3):155–239, 2006. ISSN 1567-2190. doi: 10.1561/

0100000006. URL http://dx.doi.org/10.1561/0100000006.

[14] Angelos Dassios and Hongbiao Zhao. Exact simulation of Hawkes process with exponentially decaying intensity. *Electronic Communications in Probability*, (18):1–13, 2013. doi: 10.1214/ECP.v18-2717.

[15] John M. Beggs and Dietmar Plenz. Neuronal avalanches in neocortical circuits. *Journal of Neuroscience*, 23(35):11167–11177, 2003. ISSN 0270-6474, 1529-2401. doi: 10.1523/JNEUROSCI.23-35-11167.2003. URL https://www.jneurosci.org/content/23/35/11167.

[16] Woodrow L. Shew, Hongdian Yang, Thomas Petermann, Rajarshi Roy, and Dietmar Plenz. Neuronal avalanches imply maximum dynamic range in cortical networks at criticality. *The Journal of Neuroscience*, 29(49):15595, 2009. doi: 10.1523/JNEUROSCI.3864-09.2009. URL https://pmc.ncbi.nlm.nih.gov/articles/PMC3862241/.

[17] Samora Okujeni and Ulrich Egert. Structural Modularity Tunes Mesoscale Criticality in Biological Neuronal Networks. *Journal of Neuroscience*, 43(14):2515–2526, 2023. ISSN 15292401. doi: 10.1523/JNEUROSCI.1420-22.2023.

[18] V Pasquale, P Massobrio, L L Bologna, M Chiappalone, and S Martinoia. Self-organization and neuronal avalanches in networks of dissociated cortical neurons. *Neuroscience*, 153(4): 1354–69, 2008. ISSN 0306-4522. doi: 10.1016/j.neuroscience.2008.03.050. URL http://www.ncbi.nlm.nih.gov/pubmed/18448256.

[19] Antonio J. Fontenele, Nivaldo A.P. de Vasconcelos, Thaís Feliciano, Leandro A.A. Aguiar, Carina Soares-Cunha, Bárbara Coimbra, Leonardo Dalla Porta, Sidarta Ribeiro, Ana João Rodrigues, Nuno Sousa, Pedro V. Carelli, and Mauro Copelli. Criticality between cortical states. *Physical Review Letters*, 122(20):208101, 2019. doi: 10.1103/PhysRevLett.122.208101. URL https://link.aps.org/doi/10.1103/PhysRevLett.122.208101.

[20] P. Golshani, J.T. Goncalves, S. Khoshkhoo, R. Mostany, S. Smirnakis, and C. Portera-Cailliau. Internally Mediated Developmental Desynchronization of Neocortical Network Activity. *Journal of Neuroscience*, 29(35):10890, 2009. doi: 10.1523/JNEUROSCI.2012-09.2009. URL http://neuro.cjb.net/cgi/content/abstract/29/35/10890.

[21] Laura Mòdol, Monika Moissidis, Martijn Selten, Fazal Oozeer, and Oscar Marín. Somatostatin interneurons control the timing of developmental desynchronization in cortical networks. *Neuron*, 112(12):2015–2030.e5, 2024. ISSN 10974199. doi: 10.1016/j.neuron.2024.03.014.

[22] Ivan Dornic, Hugues Chaté, and Miguel A. Muñoz. Integration of langevin equations with multiplicative noise and the viability of field theories for absorbing phase transitions. *Phys. Rev. Lett.*, 94:100601, Mar 2005. doi: 10.1103/PhysRevLett.94.100601. URL https://link.aps.org/doi/10.1103/PhysRevLett.94.100601.

[23] Jens Wilting and Viola Priesemann. Inferring collective dynamical states from widely unobserved systems. *Nature Communications*, 9(1):2325, 2018. ISSN 2041-1723. doi: 10.1038/s41467-018-04725-4. URL https://www.nature.com/articles/s41467-018-04725-4.

[24] Paul Manuel Müller, Gadi Miron, Martin Holtkamp, and Christian Meisel. Critical dynamics predicts cognitive performance and provides a common framework for heterogeneous mechanisms impacting cognition. *Proceedings of the National Academy of Sciences*, 122 (14):2017, apr 2025. ISSN 0027-8424. doi: 10.1073/pnas.2417117122. URL http://www.pnas.org/lookup/suppl/doi:10.1073/pnas.2216830120/-/DCSupplemental.https://doi.org/10.1073/pnas.2216830120https://pnas.org/doi/10.1073/pnas.2417117122.

[25] Christian Meisel, Andreas Klaus, Vladyslav V. Vyazovskiy, and Dietmar Plenz. The interplay between long- and short-range temporal correlations shapes cortex dynamics across vigilance states. *Journal of Neuroscience*, 2017. ISSN 0270-6474, 1529-2401. doi: 10.1523/JNEUROSCI.0448-17.2017. URL https://www.jneurosci.org/content/early/2017/09/25/JNEUROSCI.

0448-17.2017.
